# TRα1 mutant suppresses KLF9 to cause endometrial metaplasia with ectopic IL-33 expression leading to uterine fibrosis and infertility

**DOI:** 10.1038/s41598-025-86848-5

**Published:** 2025-01-31

**Authors:** Elijah Edmondson, Takahito Kimura, Eunmi Hwang, Minjun Kim, Andrew Warner, Yuelin Zhu, Li Zhao, Yanlin Yu, Xuguang Zhu, Maria Hernandez, Noemi Kedei, Sheue-yann Cheng

**Affiliations:** 1 Frederick National Laboratory for Cancer Research, Frederick, MD USA; 2 National Cancer Institute, National Institutes of Health, Bethesda, USA; 3 Gene Regulation Section, Laboratory of Molecular Biology, National Cancer Institute, National Institutes of Health, 37 Convent Dr, Room 5128, Bethesda, MD 20892-4264 USA

**Keywords:** Thyroid hormone receptor α1, Female reproduction, Resistance to thyroid hormone, KLF9, Spatial transcriptomics, Endometrial metaplasia, Infertility, Genetics, Diseases, Endocrinology

## Abstract

Thyroid hormone receptors (TRs) mediate the genomic actions of thyroid hormone. Mutations of THRA gene cause a human disease known as resistance to thyroid hormone (RTHα). We created a mouse model expressing a dominant negative mutated TRα1 (Thra1^PV/+^ mice) that exhibits growth retardation, bone abnormalities, constipation, and anemia, as found in RTHα patients. In addition, female Thra1^PV/+^ mice exhibit decreased fertility. In the present study, we aimed to characterize the molecular events leading to infertility. Histologically, there was progressive uterine atrophy in Thra1^PV/+^ mutant mice, characterized by squamous metaplasia of the endometrial mucosa and endometrial fibrosis. RNA-seq analysis of laser-captured micro-dissected endometrium and spatial transcriptomics revealed a key role for Krüppel-like factor (Klf9), a directly-regulated TR target gene, in normal endometrial differentiation. Klf9 was suppressed in the endometrium of mice harboring mutated TRα1 and pathway analysis revealed that deficient Klf9 signaling was associated with squamous differentiation, consistent with the endometrial metaplasia observed histologically. Further, we showed that this metaplastic endometrial mucosa was the source of ectopic IL-33, which was associated with increased T-cell infiltrates, destruction of glands, and endometrial fibrosis. Our studies provide new insights to understand uterine epithelial morphogenesis and how thyroid dysfunction could lead to female infertility.

**Supplementary Information:**

The online version contains supplementary material available at 10.1038/s41598-025-86848-5.

## Introduction

Thyroid hormones (TH) are known to affect female reproductive function^1,2^. TH modulate the development, differentiation, and metabolism of the ovary and uterus and abnormal thyroid status – hypothyroidism or hyperthyroidism – are associated with reproductive abnormalities, such as menstrual irregularity, infertility, poor pregnancy outcomes, and gynecological disorders^1,2^. These observations indicate a complex molecular interplay between the biological activity of TH and reproductive function; however, the molecular mechanisms underlying reproductive abnormalities with thyroid dysfunction are incompletely understood^1,2^.

Thyroid hormone receptors (TR) are central to the molecular actions of TH. TRs are TH-dependent transcription factors that mediate the genomic actions of TH in differentiation, development, and maintenance of metabolic homeostasis. Humans have two TR genes, *THRB* and *THRA*, which are located on different chromosomes and encode three major TH binding receptors: TRβ1, TRβ2, and TRα1. These TH receptors share high sequence homology in the DNA binding domain and hormone binding domain, but differ in the length and amino acid sequences in the amino-terminal A/B domains. Expression of these TR isoforms is tissue-dependent and developmentally regulated^3–5^ and there are documented TR isoform-specific functions as well as redundant functions^6^. These are regulated by different types of TH response elements on the TH target genes, as well as by a host of co-repressors and co-regulators. In addition, TR can mediate actions via non-genomic pathways^7–9^.

Mutations of the *THR* genes are known to cause human disease. Despite high sequence homology in the DNA and hormone binding domains, distinct in vivo molecular actions of TRβ and TRα1 produce unique clinical manifestations in patients. *THRB* mutations, known as resistance to thyroid hormone β (RTHβ), present with elevated serum TH, non-suppressible thyroid stimulating hormone (TSH), and other symptoms, including slow growth, hearing loss, and attention deficit–hyperactivity disorder^10^. In contrast, mutations of the *THRA* gene, known as resistance to thyroid hormone α (RHTα), produce near-normal thyroid function tests, but patients develop an array of distinct abnormalities compared to RTHβ, including growth retardation, delayed bone development, constipation, and erythroid disorders^11–13^. Much has been learned about the molecular actions of TRβ mutants through extensive studies in cultured cells and mouse models^10,14,15^. However, relatively little is known about RTHα mutations, which were first reported in 2012 and 2013 ^11,12,16^, 23 years after the identification of causative mutations of the *THRB* gene in RTHβ^16^.

Prior to the discovery of patients with THRA mutations, three *THRA* gene knock-in mouse models were created to explore the in vivo molecular actions of TRα1. A knock-in mutant mouse expressing a dominant negative TRα1 mutant (R384C) exhibits enhanced basal metabolism, increased sympathetic outflow, and a lean phenotype, reversible with T3-treatment^17^. Another knock-in mutant mouse expressing a dominant negative TRα1 mutant (P384H) exhibits visceral adiposity and impaired catecholamine-stimulated lipolysis^18^. Mice expressing a potent dominant negative C-terminal frame-shifted TRα1 mutant (TRα1PV; *Thra1*^*PV/+*^ mice) are dwarfs that show delayed bone development, erythroid disorders, and intestinal abnormalities^15,19–22^. Subsequently, a TRα1 mutant mouse expressing TRa1L400R, after CRE/LoxP-mediated DNA recombination, was generated. Early expression of the dominant negative mutant affects postnatal development and adult homeostasis^23^. Notably, dominant negative TRα1 mutants with different mutation sites differ in the phenotypic manifestations, suggesting diverse actions of TRa1 mutants. These in vivo studies facilitated the identification of patients with mutations of the *THRA* gene and have advanced the understanding of the in vivo actions of TRα1 mutants^11,12^.

The phenotypic complexity produced in mice with TRa1 mutations, including infertility, presents an opportunity to better understand the interplay between thyroid hormone signaling and reproductive disease. Infertility in *Thra1*^*PV/+*^ mice was driven by uterine pathology characterized by endometrial squamous metaplasia with transcriptional perturbations that included suppression of Krüppel-Like factor 9 (*Klf9*), a key transcriptional regulator for squamous differentiation^24^ and endometrial differentiation^25,26^. The endometrial squamous metplasia in *Thra1*^*PV/+*^ mice served as a source for ectopic IL-33 expression, normally expressed in vaginal squamous mucosa, but not uterus^27^. Uterine IL-33 dysregulation has also been linked reproductive pathology in women^28^. Our studies reveal a link between TRa1 mutation, deficient uterine Klf9 signaling, endometrial metaplasia, and IL33-induced uterine pathology.

## Results

### *Thra1*^*PV*/+^ mice develop progressive uterine atrophy, endometrial squamous metaplasia, and uterine fibrosis

**Fig. 1:**
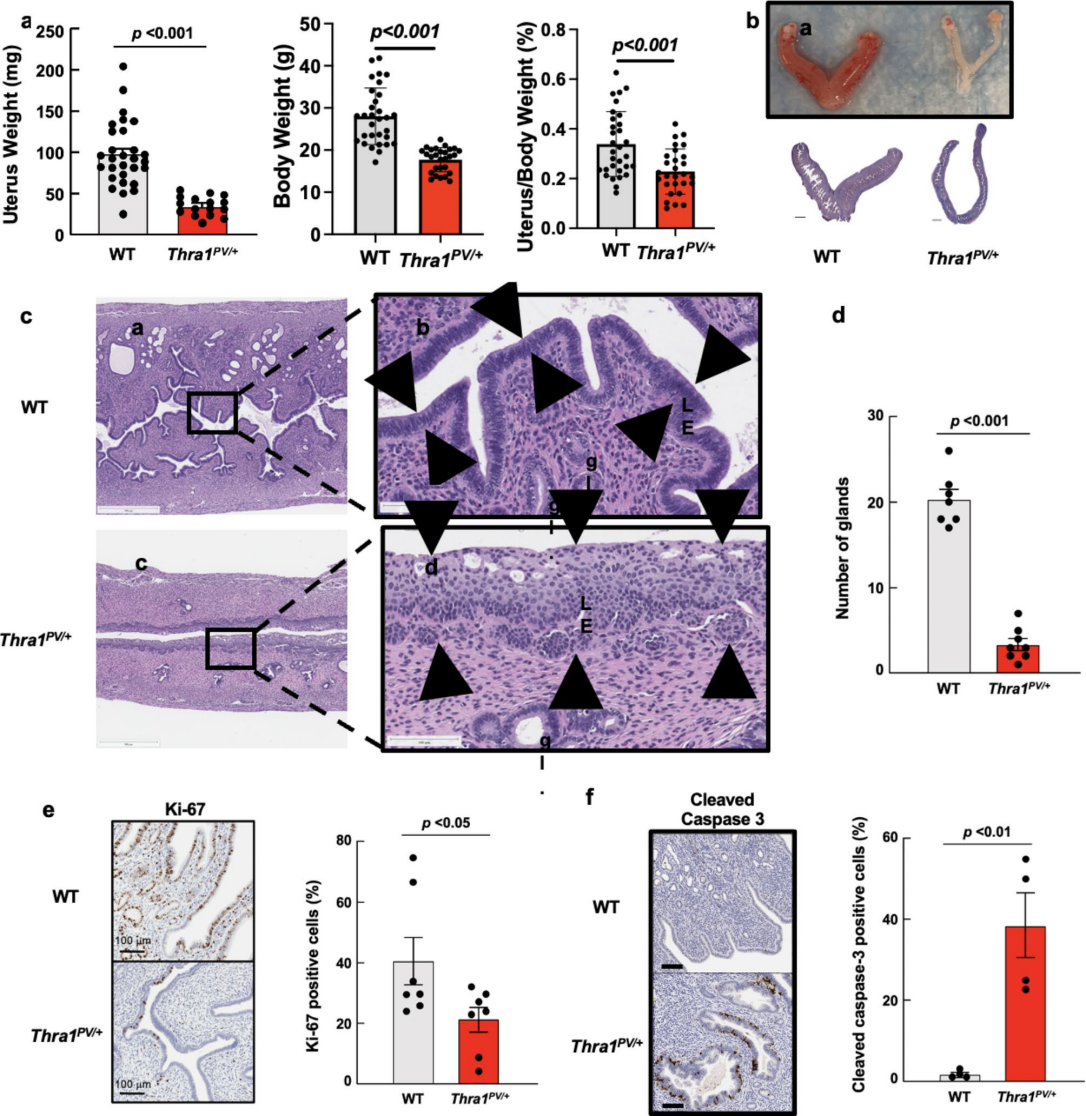
Uterine atrophy in*Thra1*^*PV/+*^ mice is characterized by decreased uterine weight, size, decreased Ki-67 staining, and increased cleaved caspase-3 staining in the mucosa. (**a**) Uterus of WT and *Thra1*^*PV/+*^ mice were dissected and weighed (*n* = 31 for WT, *n* = 16 for *Thra1*^*PV/+*^ mice). (**b**) Gross and histologic images of uterus from WT and *Thra1*^*PV/+*^ mice at 6 months of age (**c**) Histology of wildtype and *Thra1*^*PV/+*^ mutant uterus; solid arrow heads outline uterine mucosa at 6 months of age. (**d**) Quantification of the number of endometrial glands per mm^2^. (**e**) Comparison of Ki-67 proliferative index in WT and mutant endometrium. **(f)** Comparison of cleaved caspase-3. Error bars represent means *±* SEM. *p* < 0.05 *; *p* < 0.01 **; *p* < 0.001 ***.

Comparison of uterus weight between wild-type (WT) and *Thra1*^*PV*/+^ mice showed a 65% reduction in mutant mice (Fig. 1a). When controlling for the decreased body weight of *Thra1*^*PV*/+^ mice, the relative uterus weight was still decreased compared to WT mice (Fig. 1a). The mutant uterus was grossly decreased in size and pale (Fig. 1b). We next examined uterine sections histologically from estrous cycle-matched females to characterize the morphologic changes associated with reduced uterus weight. The uterine lining in WT mice was composed of a simple columnar epithelium that branched into tubular glands within the endometrium (Fig. 1c). In contrast, the mutant endometrial lining was attenuated and often composed of stratified squamous cells with markedly reduced glands (Fig. 1c). The reduction in number of glands was extensive, with an 82% decrease in the number of glands per mm^2^ in the endometrium of mutant mice compared to WT (Fig. 1d).

The abnormally differentiated luminal epithelium in *Thra1*^*PV*/+^ mice was characterized by a multifocal replacement of the single layer of columnar cells by stratified squamous cells (Fig. 1c). The squamous metaplasia often affected greater than 50% of the endometrial epithelium and affected all regions of the uterine horn similarly.

The proliferative index of the endometrial epithelium was quantified with immunohistochemical (IHC) staining for Ki-67, which showed decreased staining in the endometrial luminal epithelium and glands of the mutant uterus (Fig. 1e). In addition to decreased proliferation, increased apoptosis was also observed in *Thra1*^*PV*/+^ mice, as indicated by increased cleaved caspase-3 positive glands as compared with WT mice (Fig. 1f). These data demonstrate that the reduced uterus weight in *Thra1*^*PV*/+^ mice was associated with decreased proliferation and increased apoptosis in the endometrial epithelium.

**Fig. 2:**
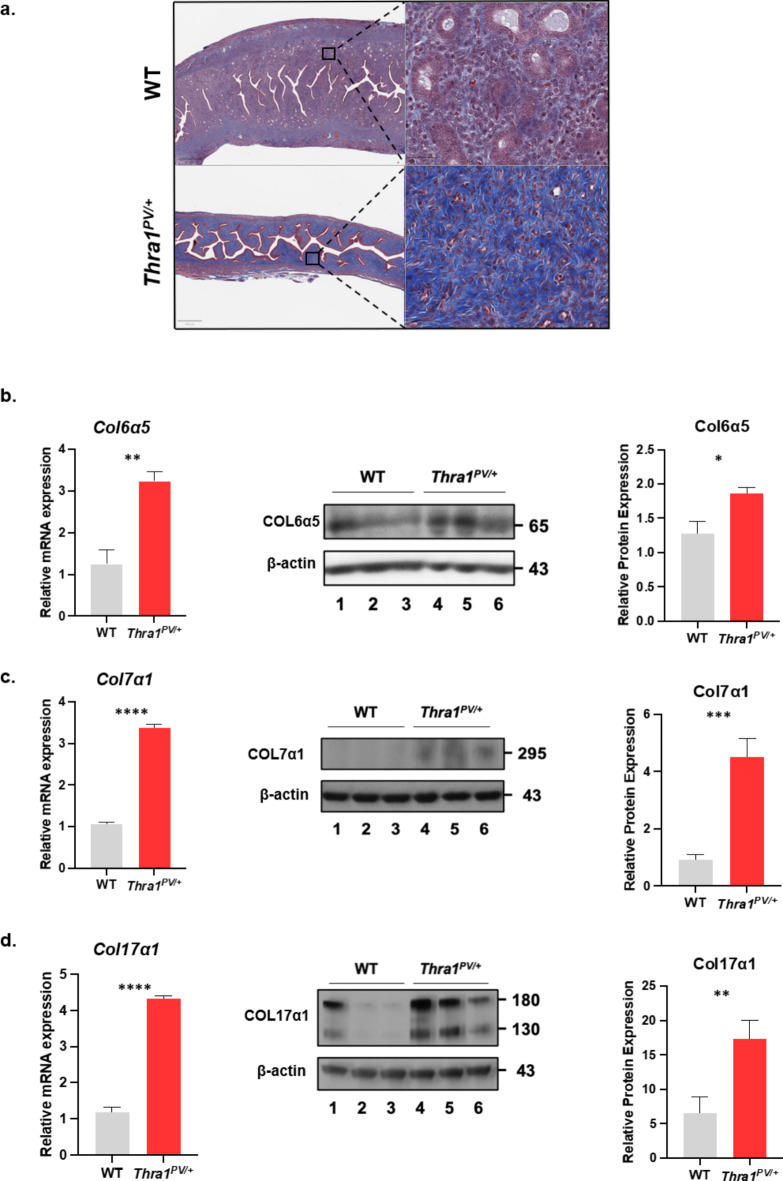
Endometrial fibrosis in *Thra1*^*PV/+*^ mice. (**a**) Sections of WT and *Thra1*^*PV/+*^ uterus at 6 months of age stained with Masson’s Trichrome showed increased fibrosis in mutant endometrium (**b-d**) The expression of three collagen genes (*Col6a5*, *Col7a1*, and *Col17a1*) was determined by qPCR and was shown to be elevated in the uterus of *Thra1*^*PV/+*^ mice (age: 5–7 months) as compared with WT uterus (age: 4–6 months) (B-a, b, and c, respectively). Increased protein levels of three collagens (COL6a5, COL7a1, and COLl17a1) was demonstrated by western blot (WB) Values are means *±* SEM from duplicated runs, each with 5 WT and 5 mutant mice for C-II-a, and C-II-b. Values are means *±* SEM from duplicated runs, each with 3 WT and 3 mutant mice. *p* < 0.05 *; *p* < 0.01 **; *p* < 0.001 ***; *p* < 0.0001 ****.

Loss of endometrial glands and increased endometrial fibrosis *were observed* in mutant mice. Trichrome staining in sections of WT and mutant uterus demonstrated redundant collagen fibrils that replaced normal endometrial glands and stroma in the atrophied uterus of mutant mice. (Fig. 2a). We then carried out western blot analysis to quantify the collagen types commonly expressed in the uterus. The expression of three collagen genes, *Col6a5*, *Col7a1*, and *Col17a1*, were elevated 2.6-3.7-fold at the mRNA level in the uterus of *Thra1*^*PV*/+^ mice (Fig. 2b, c, and d respectively). Consistently, the protein levels of these collagens were also elevated in the endometrium of mutant mice (Fig. 2b, c, and d)) for Col6a5, Col7a1, and Col17a1, respectively. These results demonstrate that mutations of the *Thra* gene result in endometrial fibrosis in the mouse uterus.

### Altered global gene profiles in distinct cellular compartments of WT and mutant endometrium

The histological abnormalities observed prompted us to comprehensively evaluate transcriptional pertubations induced by mutations of the *Thra* gene at the genome-wide level. We carried out bulk RNA-seq of laser-captured micro-dissected (LCM) endometrium from WT and *Thra1*^*PV*/+^ mice. To eliminate the effects of the hormonal fluctuations during the estrous cycle, we synced both the WT mice and mutant mice at the metestrus phase. The differentially expressed gene (DEG) (Supplementary Table 1) and unsupervised hierarchical clustering (Supplementary Fig. 1a) showed distinct endometrial gene expression profiles for WT and mutant mice. We detected 592 DEG (*p* < 0.05) using bulk RNAseq on LCM capture endometrium. It is of interest to note that the up-regulated genes (489 genes) were nearly 5-fold higher than the down-regulated genes (102 genes).

The complex histologic changes in the mutant endometrium described above suggest that the DEG could act within the context of micro-anatomical compartments to mediate abnormal phenotypic manifestations. We therefore used the GeoMx Digital Spatial Profiler^29^ Whole Transcriptome Atlas to spatially map genes differentially expressed between WT and mutant in distinct anatomical regions of the endometrium, including luminal epithelium, glands, and endometrial stroma.

**Fig. 3:**
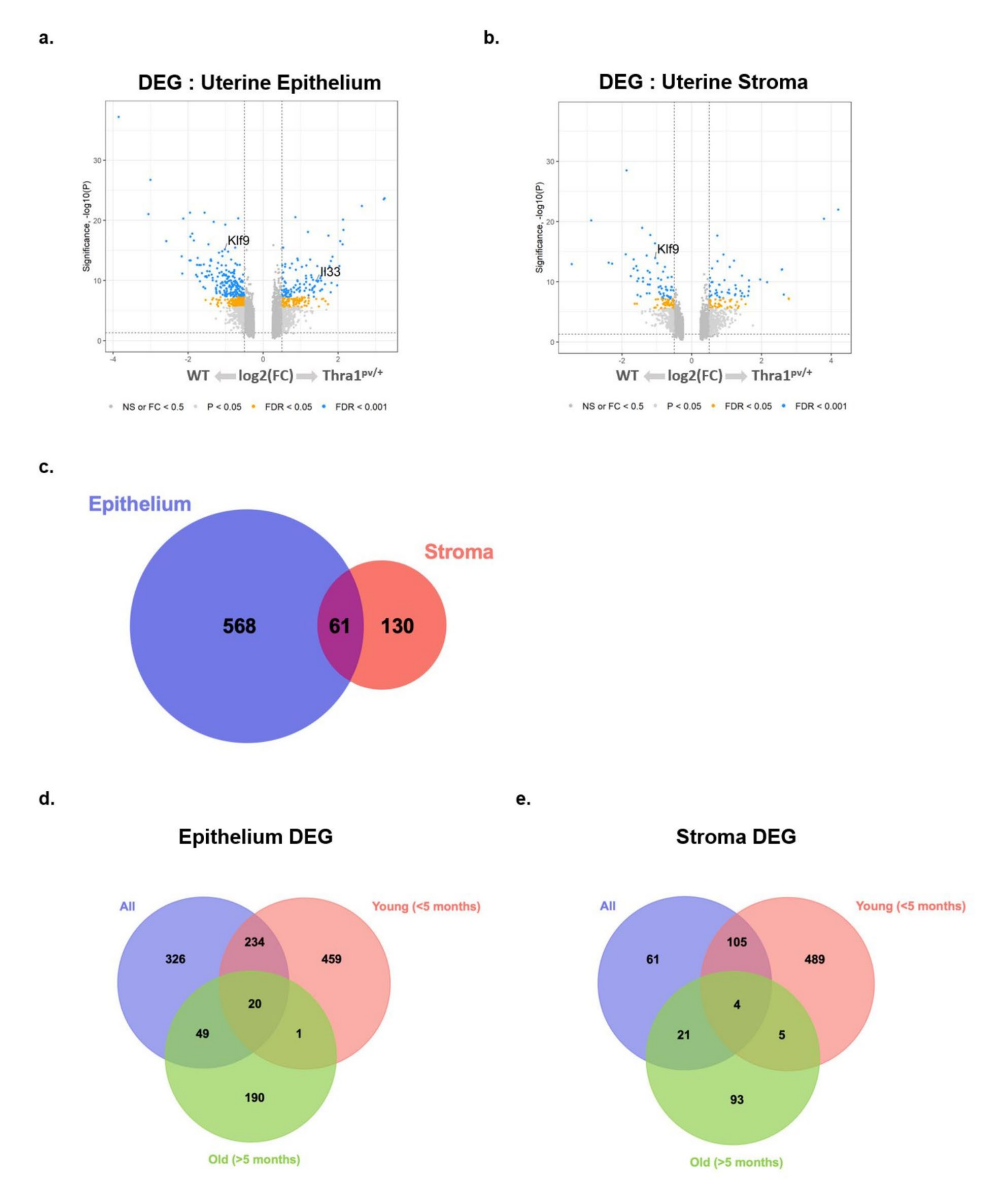
Distinct gene expression in the epithelium and stroma of WT and*Thra1*^*PV/+*^ endometrium by spatial profiling. (**a**) Differentially expressed genes (DEG) of the uterine epithelium and (**b**) uterine stroma. *Klf9* expression was decreased in mutant epithelium and stroma. (**c**) Distinct gene expression patterns between epithelium and stroma were observed; 61 DEG were common between these two spatial compartments. (**d**) Venn diagrams show the limited number of overlapping genes between young (< 5 months) and old (> 5 months) in both the epithelium (**d**) and stroma (**e**).

Spatial transcriptomics provided distinct gene expression patterns for uterine epithelium and stroma. Unsupervised clustering of highly variable genes revealed distinct clusters that separated by genotype, histology class (mucosa, gland, or stroma), and age (Supplementary Fig. 2). DEG patterns were detected in uterine epithelium (Fig. 3a) and stroma (Fig. 3b). As shown in Tables 1, 629 DEG were found in the epithelium; 435 genes were enriched in WT and 194 genes were enriched in mutant. Fewer DEG (*n* = 191) were detected in the endometrial stroma, with 104 enriched in WT and 87 enriched in mutant stromal tissue.

Overlap in DEG between epithelium and stroma was observed for only 61 DEG (Fig. 3c), indicating unique transcriptional programs in the epithelial and stroma compartments. Interestingly, as mice aged, a lower number of DEG were detected in epithelium (from 470 to 260 genes) and stroma (from 319 genes to 124 genes) (Table 1). The age-dependent differences in DEG can be seen in the Venn diagram (Fig. 3, d and e), in that there were only 21 overlapping DEGs between the young (< 5 months) and the old (> 5 months). Similarly, a small number of overlapping DEGs (9 genes) were found in the stroma between the young (< 5 months) and the old (> 5 months) (Fig. 3e).

**Table 1:** Comparison of differentially expressed genes (DEG) in uterine epithelium and stroma in younger and older mice.

	WT enriched genes	Thra1^PV/+^ enriched genes	Total DEGs
Epithelium	435	194	629
Stroma	104	87	191
Epithelium *2–5 months*	225	245	470
Stroma *2–5 months*	132	187	319
Epithelium *5–10 months*	59	201	260
Stroma *5–10 months*	63	60	124

### TRα1 mutation suppresses uterine KLF9 signaling, resulting in abnormal squamous differentiation of the endometrial epithelium

One DEG in the endometrium that captured our attention was the Krüppel-Like Factor 9 (*Klf9*) gene. Earlier studies have indicated that global loss of KLF9 expression, though not lethal to embryos, causes a subfertility phenotype, including decreased proliferation and increased apoptosis in endometrial glands, mucosa, and stroma cells^30^. Importantly, KLF9 is directly regulated by TH in a TR-dependent manner both in vivo and in vitro^31,32^. TRs induce Klf9 expression to regulate neurogenesis, hepatocyte proliferation, and hepatocyte differentiation^32^. Notably, KLF9 controls the proliferation of squamous cells^24^. We thus hypothesized that endometrial squamous metaplasia in *Thra1*^*PV/+*^ mice could be mediated via deficient KLF9 expression.

**Fig. 4:**
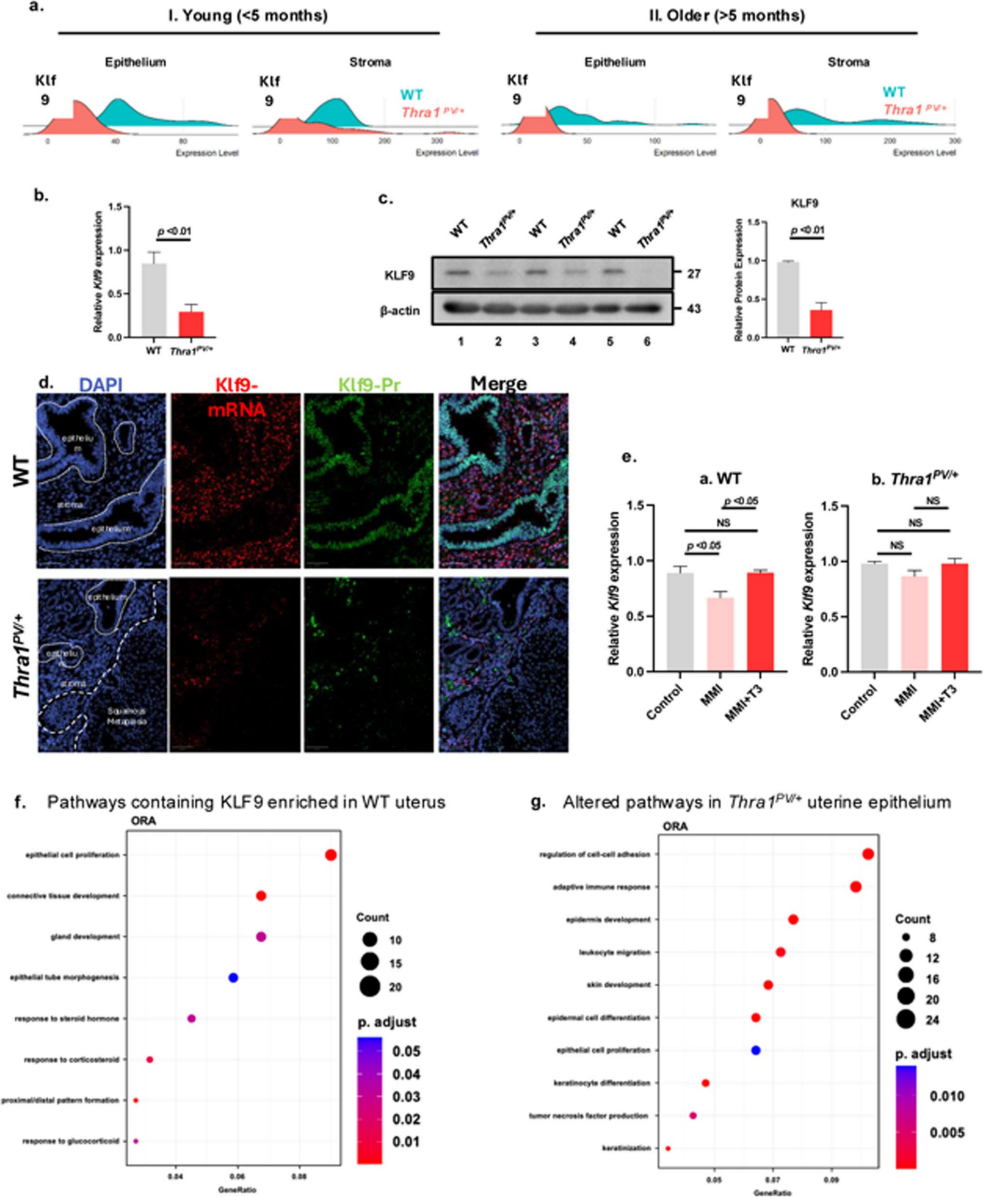
TRa1PV-suppressed KLF9 signaling alters signaling to induce endometrium abnormalities of *Thra1*^*PV/+*^ mice. (**a**). A ridge plot shows decreased *Klf9* expression in the epithelium and stroma in younger (< 5 months) and older mice (> 5 months). (**b**) The expression of the *Klf9* gene was suppressed, as determined by q/PCR using uterus from WT and *Thra1*^*PV/+*^ mice. (**c**) KLF9 protein levels were lower in the uterus of *Thra1*^*PV/+*^ mice (*n* = 3) by western blot analysis; values are means *±* SEM from duplicated runs, each with 3 WT and 3 mutant mice. (**d**) *Klf9* RNAscope and immunohistochemistry on uterine endometrium. (**e**) The expression of the.

To support this hypothesis, we analyzed the expression of the *Klf9* gene using multiple approaches. The expression of the *Klf9* gene was significantly decreased in both the *Thra1*^*PV/+*^ uterine epithelium and stroma when compared to WT mice (Fig. 4a). The expression of the *Klf9* gene was 2.4 and 3.5-fold higher than mutant in uterine epithelium and stroma, respectively. Confirmation using qPCR and WB analyses of total uterus showed a reduction of ~ 65% in the expression of *Klf9* mRNA (Fig. 4b) and protein levels in the mutant mice (Fig. 4c). To localize *Klf9* mRNA and protein in tissue sections, we multiplexed RNAscope and IHC to determine the histologic and spatial context of decreased *Klf9* expression (Fig. 4d). Increased *Klf9* mRNA was observed in WT compared to mutant, particularly in the endometrial stroma. Klf9 protein was also increased in WT, with an intense nuclear pattern in the WT epithelium compared to mutant, which had a relatively weak nuclear signal (Fig. 4d). These results further confirmed that the *Klf9* gene was expressed both in epithelium and stroma compartments and was suppressed in both compartments of mutant mice.

*Klf9* gene is regulated by T3: WT and *Thra1*^*PV/+*^ mice were rendered hypothyroid by treatment with methimazole, with or without T3 supplementation. Total RNA was extracted, and the expression of *Klf9* gene was determined by q/PCR. Values are means *±* SEM (*n* = 3). (f.) Pathway analysis from spatial transcriptomic data; significantly enriched pathways containing KLF9 pathways in WT uterus (G). Pathway over-representation analysis showing enriched pathways in *Thra1*^*PV/+*^ uterine epithelium.

Though it was reported that TR/T3 directly regulates the expression of the *Klf9* gene in the liver^32^ and in the bone marrow^31^, we also carried out an experiment to demonstrate that the *Klf9* gene was also regulated by T3 in the uterus of WT mice. We induced hypothyroidism in the WT and *Thra1*^*PV/+*^ mice by treating them with methimazole (MMI), with or without supplementation with T3. MMI treatment of WT mice decreased KLF9 protein levels, which was restored with T3 treatment (Fig. 4e-a). However, in *Thra1*^*PV/+*^ mice, T3 regulation was lost (Fig. 4e-b). We also determined the relative abundance of TRβ1 and TRα1 in the uterus by co-immunoprecipitation analysis. We found that the major TR isoform in the uterus was TRα1 (Supplementary Fig. 3a and b) and confirmed that TRα1PV protein was expressed in the uterus of *Thra1*^*PV/+*^ mice (Supplementary Fig. 3c). These results expand the target tissue to the uterus, in addition to the liver and bone marrow, in which the expression of the *Klf9* gene is regulated by TR/T3.

To assess the functional consequences of the reduced expression of the *Klf9* gene, we carried out pathway enrichment analysis of the differentially expressed genes. Gene Ontology analysis (GO) showed KLF9 signaling was extensively involved in many biological processes and molecular functions, notably epithelial cell proliferation, gland development, female pregnancy, and responses to hormones (Supplementary Table 2). Over-representation analysis (ORA) of genes enriched in WT endometrium showing top pathways were involved in epithelial cell proliferation and morphogenesis, connective tissue development, gland development, and responses to hormones (Fig. 4f). A contrasting gene expression pattern emerged in the mutant uterine epithelium; upregulated pathways in endometrial epithelium of *Thra1*^*PV/+*^ mice included pathways for keratinocyte differentiation, keratinization, and epidermis development (Fig. 4g). The identification of these altered pathways is consistent with the phenotypic manifestation of endometrial squamous metaplasia with keratinocyte morphology in the mutant mice (see Fig. 1B).

### Abnormally differentiated endometrial cells are the source of ectopic IL-33 expression in the uterus of *Thra1*^*PV*/+^ mice

IL-33 is a pleiotropic cytokine that is critical for tissue homeostasis, inflammation, and allergic responses^33^. IL-33 is expressed by a wide variety of cell types, including stratified squamous epithelia from vagina and skin^27^. IL-33 has dual functions as a both a cytokine and as a transcription factor to regulate gene expression^34^. It is thought to function as a regulator of chronic inflammatory diseases, autoimmune diseases, and fibrotic disorders^35^ and functions as an alarmin when released from necrotic and/or damaged tissues^36,37^ in the context of the female reproductive tract, IL-33 has a significant role in normal function^38,39^ and reproductive pathology^28^. The elevated IL-33 in *Thra1*^*PV/+*^ mice prompted us to further characterize its expression in the mutant endometrium.

**Fig. 5:**
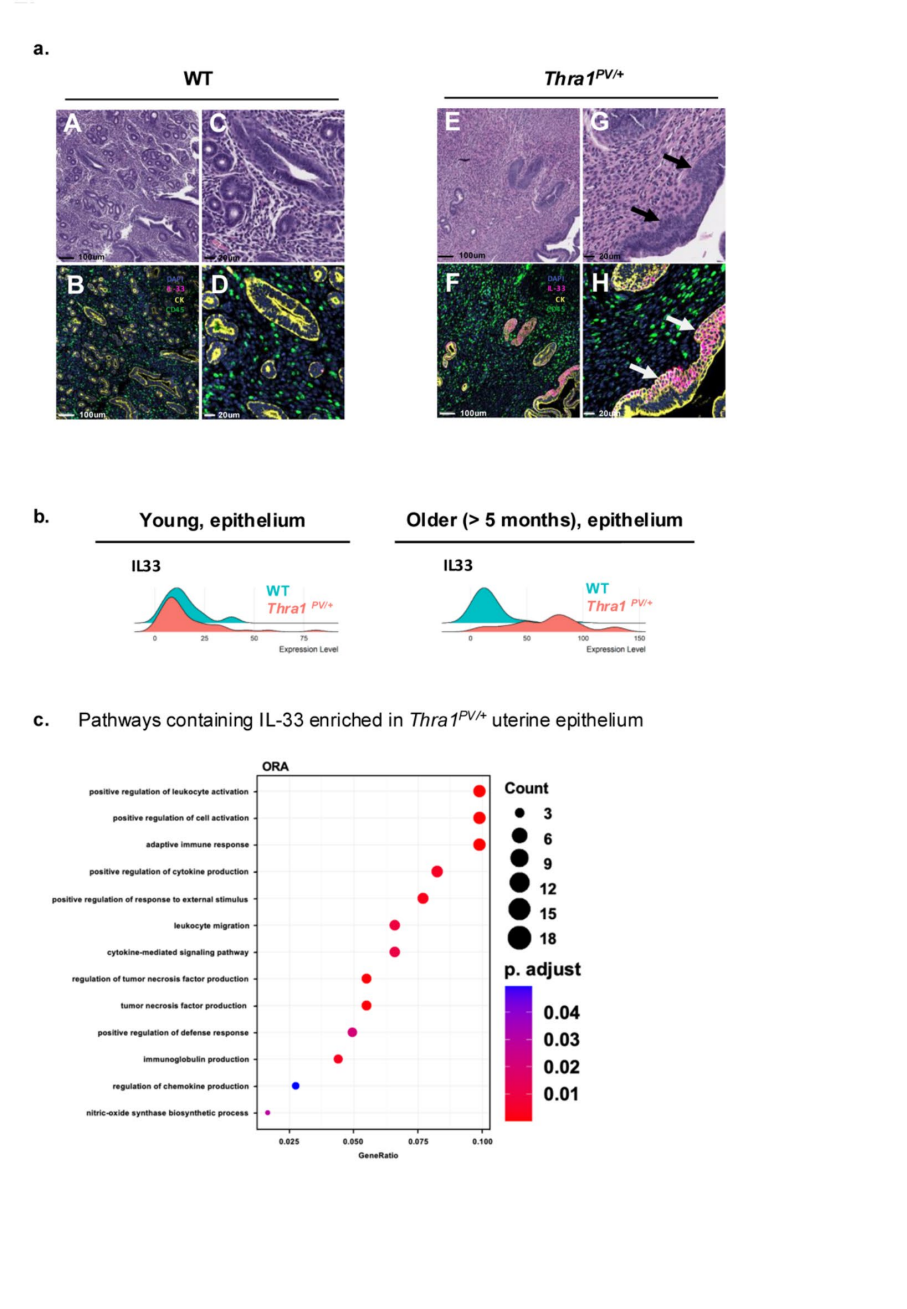
Ectopic uterine*Il-33* expression in regions of endometrial squamous metaplasia in*Thra1*^*PV/+*^ mice. (**a**) Histological comparison of the endometrium and mRNA expression of the *Il-33* gene in the WT and mutant epithelium; solid arrows mark regions of endometrial metaplasia in serial sections stained for H&E (panel G) and IL-33 RNAscope (red) and CK (yellow) and CD45 (green) immunohistochemistry. Cytokeratin (CK) and CD45 were used to label epithelium and stroma, respectively. (**b**) Ridge plot of *Il-33* gene expression in epithelium of young and older WT and *Thra1*.^*PV/+*^ mice (**c**) Enriched pathways in *Thra1*.^*PV/+*^ uterine epithelium containing IL-33.

We multiplexed RNAScope^40^ to detect *Il-33* mRNA with IHC for Cytokeratin and CD45 to localize the cellular source of *Il-33* in the mutant and WT uterus. *Il-33* expression was rare in WT endometrial epithelium (Fig. 5a, sub-panel C and D). In contrast, *Il-33* expression in *Thra1*^*PV/+*^ endometrial epithelium was substantial (Fig. 5a, sub-panel G and H). Strikingly, *Il-33* expression in *Thra1*^*PV/+*^ localized to regions of endometrial squamous metaplasia and was absent in adjacent simple columnar epithelium areas (sub-panel G, marked by solid arrows). Spatial transcriptome data revealed that *Il-33* expression was increased in older mutant mice (5–10 months of age) and was mostly within normal limits in the uterine epithelium from younger mice (Fig. 5b). As WT mice aged, there were no marked endometrial changes and *Il-33* expression was not elevated (Supplementary Fig. 4a and c). However, in the endometrium of aged mutant mice, glands were progressively less common and endometrial squamous metaplasia was prominent (Supplementary Fig. 4b and d). Again, *Il-33* expression consistently localized with endometrial squamous metaplasia (Supplementary Fig. 4b and d). These results demonstrate that *Thra1*^*PV/+*^ mice have persistent *Il-33* expression in the uterine mucosa associated with squamous metaplasia.

Although *Il-33* expression was rare in WT uterus, intense *Il-33* expression is observed diffusely in the stratified squamous epithelium of the cervix and vagina (Supplementary Fig. 5). This is consistent with previous reports, which demonstrated that IL-33 is highly expressed in mouse epithelial barrier tissues, including vaginal mucosa and skin^27^. *Il-33* expression was also observed in the cervix and vagina of *Thra1*^*PV/+*^ mice (Supplementary Fig. 5), but unlike WT mice, intense *Il-33* expression was also observed in the uterine endometrium. Given the intense *Il-33* expression in normal squamous epithelial cells of the cervix and vagina and the aberrant expression of *Il-33* in regions of endometrial squamous metaplasia of mutant mice, we conclude that this *Il-33* expression is ectopic to the uterus.

### *Il-33* Over-expression is associated with altered immune landscape in *Thra1*^*PV/*+^ mice

Using transcriptomic and pathway analysis, we next analyzed the IL-33 mediated pathways by ORA and found that the top pathways were enriched with immune responses such as leucocyte activation and migration, adaptive immune response, defense responses, and immunoglobulin, chemokine, and tumor necrosis factor production (Fig. 5c). In addition, we carried out Ingenuity Pathway analysis on DEG from RNA-seq on laser-captured endometrium and total uterus, which indicates extensive upregulation of immune signaling in *Thra1*^*PV/+*^ mutant mice (Supplementary Fig. 1b). Consistently, we found that among the top 15 pathways identified, 12 pathways were related to lymphocyte functions and signaling, including as B-cell development, B-cell receptor signaling, and other immune responses (Supplementary Fig. 1b).

**Fig. 6:**
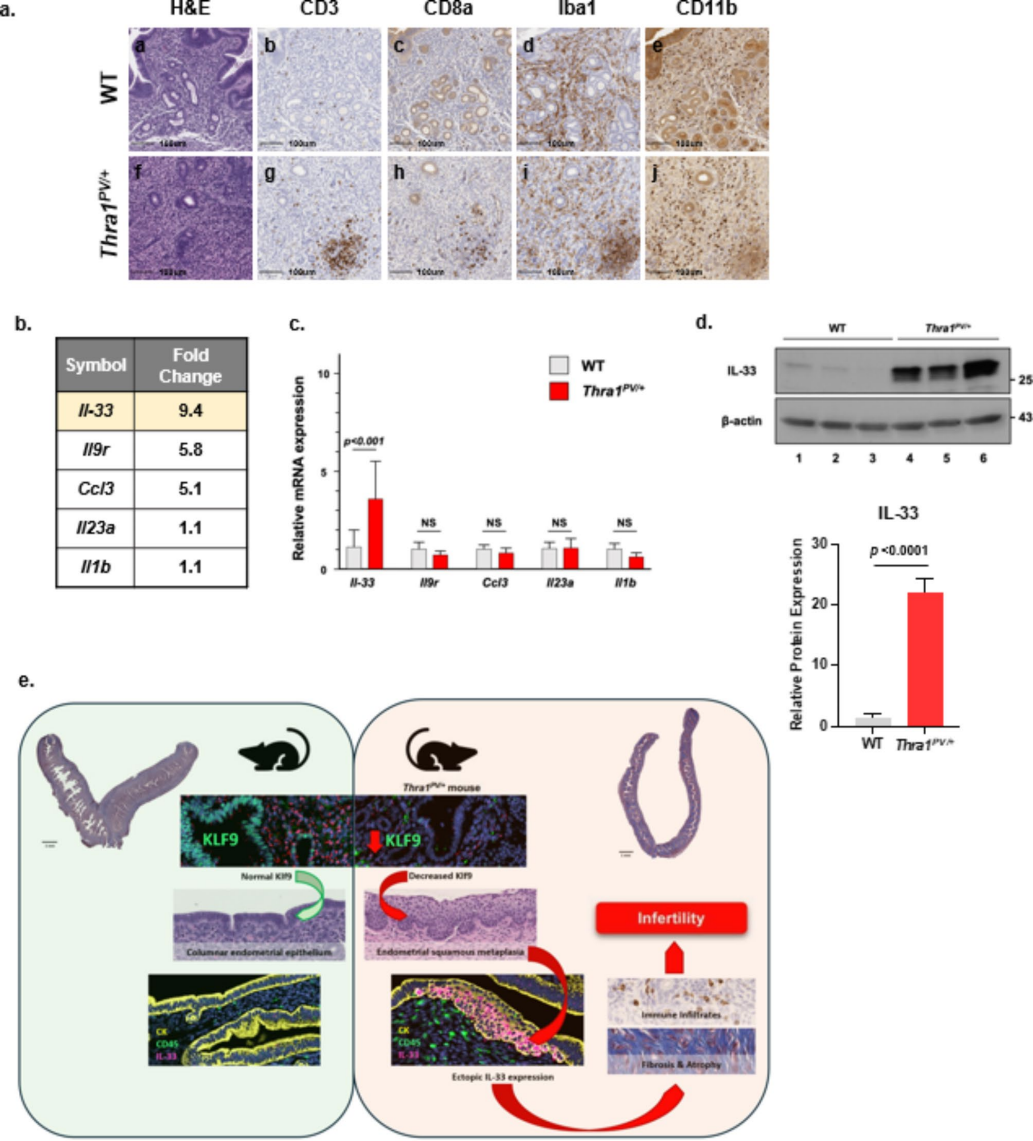
Altered immune landscape in the endometrium of*Thra1*.^*PV/+*^ mice. (**a**) Infiltration of immune cells in the endometrium of *Thra1*.^*PV/+*^ mice. Immunohistochemistry for CD3, CD8a, Iba1, and CD11b. (**b**) Changes in the expression of major cytokines determined by RNA-seq. (**c**) Real-time PCR analysis of cytokines from total RNA of WT and *Thra1*.^*PV/+*^ uterus (**d**) Comparison of IL-33 protein levels in the uterus of WT and *Thra1*.^*PV/+*^ mice by western blot analysis; values are means *±* SEM from duplicated runs, each with 3 WT and 3 mutant mice. (**e**) Graphic representation of a molecular model of the events leading to infertility of *Thra1*^*PV/+*^ mice.

To characterize the immune cell infiltrates in mutant endometrium, we used immunohistochemistry to quantify CD3, CD8a, Iba1, and CD11b positive cells in the endometrium of WT and mutant mice. In *Thra1*^*PV/+*^ endometrial stroma, we found increased CD3 and CD8a positive T-cells (Fig. 6a) and similar numbers of Iba1 and CD11b positive immune cells (Fig. 6a).

The infiltration of the immune cells into the endometrium of mutant mice prompted us to further evaluate the differential expression of cytokine genes. We identified five significantly differentially expressed cytokine genes with > 1.1-fold of change (Fig. 6b). However, quantitative real-time PCR analysis of the five genes only validated the elevated expression of the *Il-33* mRNA in the mutant mice (Fig. 6c). Western blot analysis on uterus tissue further validated the high elevation of IL-33 at the protein levels in mutant uterus (Fig. 6d).

In WT mice, normal Klf9 expression maintains epithelium development, proliferation and functions (Fig. 6e). In *Thra1*^*PV/+*^ mice, suppression of uterine Klf9 alters endometrial cell differentiation, culminating in endometrial squamous metaplasia. These abnormal metaplastic cells serve as an ectopic source of high levels of the alarmin, IL-33, in the uterine endometrium (Fig. 6e). There is a cascade of immune responses in the *Thra1*^*PV/+*^ uterus, resulting in immune cell infiltrates, endometrial fibrosis, uterus atrophy, and infertility (Fig. 6e).

## Discussion

The impact of thyroid dysfunctions on female reproduction has been long recognized^2^. Thyroid disorders can affect menstruation, ovulation, embryo implantation, and development of reproductive organs. While the association of thyroid disorders with female infertility is evident, the underlying mechanisms are less clear. Hypotheses were put forward to suggest that deleterious thyroid disorders on female reproduction were mediated by defective signaling of TRs. To this end, the expression of TRs was demonstrated in the luminal epithelium, glands, stroma, and myometrium of human and other animal uteri^41–43^. However, how the expressed TRs acted to affect female infertility has not been elucidated. The observations that *Thra1*^*PV/+*^ mice are infertile provided an opportunity to dissect the pathogenic actions of the TRα1 mutant to shed light on the in vivo molecular actions of TRα1 on female reproduction.

In the *Thra1*^*PV/+*^ mice, we found atypical endometrial cell differentiation characterized by squamous metaplasia, loss of endometrial glands, and fibrosis in the mutant endometrium. Importantly, the extensive metaplasia in the endometrial epithelium served as a source of high levels of Il-33, which altered the immune milieu resulting in infiltration of T-cells in the mutant mucosa. These histologic lesions caused by the TRα1 mutant clearly diminish the capacity of the uterus to function normally and to be receptive for embryo implantation and development.

Examination of bulk RNA-seq from laser-captured uterine endometrium revealed that the TRα1 mutant brought on immune changes, as evidenced by the pathway analysis (Supplementary Fig. 1b). A subsequent deeper spatial transcriptomic analysis using GeoMx DSP demonstrated distinct transcriptional alterations caused by the TRα1 mutant in endometrial epithelium and stroma. One down-regulated gene that captured our attention was *Klf9*, a transcription factor that is responsive to thyroid hormone, regulates uterine endometrial differentiation^26,44^ and negatively regulates squamous differentiation^24^. We found that the *Klf9* was expressed in the WT epithelium and stroma, lost in *Thra1*^*PV/+*^ mice, and was positively regulated by TH (Fig. 4e). Previous studies have shown that the *Klf9* gene is directly up-regulated by TR/TH to modulate hematopoiesis^31,32^ and that TR induces the *Klf9* gene expression in hepatocytes and pluripotent stem cells^32^. These reports, together with our studies, demonstrate that the *Klf9* gene is a downstream target of TR/TH signaling and mutations of TRα1 (PV) led to suppression of the *Klf9* gene in the mutant endometrial mucosa.

Decreased Klf9 signaling led to transcriptional perturbations and resulted in abnormal uterine differentiation, as shown by DEG and pathway analysis. Pathways containing KLF9, which were up-regulated in WT and lost in mutant endometrium, included uterine gland development, epithelial tube morphogenesis, and hormone response pathways (Fig. 4f). In contrast, over-represented pathways in *Thra1*^*PV/+*^ mice, characterized by Klf9 loss, included keratinization, keratinocyte differentiation, and epidermal cell differentiation (Fig. 4g), consistent with the observation of squamous metaplasia in mutant epithelium. Taken together, these findings indicate that loss of uterine Klf9 signaling results in abnormal endometrial squamous differentiation.

*Il-33* is highly expressed in mouse epithelial barrier tissues, including vaginal squamous mucosa^27^ as we showed in Supplementary Fig. 5. *Il-33* is not expressed at high levels in normal uterine endometrium; however, regions of endometrial squamous metaplasia in mutant mice serve as an ectopic source of *Il-33* in the uterus (Fig. 5a). In tissues where constitutively expressed, IL-33 acts as a tissue-barrier “Alarmin” where it is passively and rapidly released from damaged cells^37^. Given that IL-33 is ectopically expressed in the dynamic uterine mucosa, which undergoes cyclic sloughing, the release of endometrial IL-33 likely amplifies immune responses withing the endometrium, leading to destruction of glands and endometrial fibrosis (Fig. 2).

The causes of female infertility are complex, involving defective reproductive organ structure (e.g., ovary, oviduct/fallopian tube, and uterus), functional disorders (e.g., inefficient ovulation), endocrine dysregulation (e.g., estrogen-, progesterone-, and thyroid hormone receptor signaling), and pathogenic conditions, such as endometriosis or cancer. In the *Thra1*^*PV/+*^ mouse, we noted no significant differences in ovarian structure in mutant mice (Supplementary Fig. 6b); although ovary tissues were generally smaller in mutant mice (Supplementary Fig. 6a), they contained similar microanatomic structures, including ovarian follicles and corpora lutea, without significant histologic lesions. It is also notable that we found no apparent changes in either of the receptor protein levels between WT and mutant mice, as shown by IHC staining of both receptors (Supplementary Fig. 7a and c). In addition, we extracted endogenous estrogens and progesterone from WT and mutant uteri and found a higher estrogens and progesterone in the in the mutant mice (Supplementary Fig. 7b and d respectively). These findings suggest that the abnormal phenotypes detected in the uterus of *Thra1*^*PV/+*^ mice were most likely not mediated by deficient estrogen receptor or progesterone receptor signaling, but mainly due to aberrant actions of TRα1 mutants.

The first RTHα patients were identified in 2012 ^11,12^, 11 years after the *Thra1*^*PV/+*^ mice were reported^15^. It was interesting to find that RTHα patients exhibit similar phenotypes of retarded growth and delayed bone development, as found in *Thra1*^*PV/+*^ mice^11,12,15^. Remarkably, the C-terminal mutated sequences of TRα1PV (marked by bold and underlined, 398-PPFVLGSV**RGL**D-409) resemble those found in the truncated C-terminal sequence in two RTHα patients (398-PPTLP**RGL**-405)^45^. Subsequent characterization also found that *Thra1*^*PV/+*^ mice display erythroid disorders and constipation, as found in RTHα patients^20–22,46,47^. These findings show that *Thra1*^*PV/+*^ mice could be used to uncover other pathogenic actions of TRα1 mutants. In the present studies, we found that mutations of TRα1 cause infertility in female mice due to a defective uterus. Recently, a germline transmittable zebrafish model of RTHα (*thrab 1-bp ins*^*m/m*^ fish) also showed female infertility due to failure to release eggs during oviposition after sexual maturation. This spawning failure was because of oviductal blockage at the genital papilla^48^. Though the abnormal phenotypic manifestations differed in these two species (mice and zebrafish), the outcome of female infertility is identical, suggesting that the pathogenic actions of the TRα1 mutant in female infertility is conserved across these two species. Up to 2019, 26 cases of RTHα cases have been reported in humans^49^. Among these patients, 50% were female. However, it is unknown whether these female patients suffer from infertility. Regardless, the present studies uncover a novel pathogenic action of TRα1 mutants and provide new insights on the mechanisms of TH actions in female reproduction.

## Materials and methods

### Animals and treatment

All methods were carried out in accordance with relevant guidelines and regulations. All experimental protocols were approved by National Cancer Institute Animal Care and Use Committee (NCI ACUC approved animal study protocol #LMB-036). *Thra1*^*PV/+*^ mice were generated as described^15^ which we continued to breed the line for our studies. All *Thra1*^*PV/+*^ mice used in the present studies were fed with NIH 31 autoclavable Teklad laboratory animal diets from Envigo (Indianapolis, IN, USA). Mice were estrous-cycle matched using MK-10 Estrous Cycle Monitor (Fine Science Tools, Inc, California). The mice were placed into soiled male caging to initate synchronization of mice. Females were then placed into a new soiled male caging 2 days later and monitored starting on Day 3 with the estrous meter and by visual inspection of the vaginal opening. Mice were determined to be in metestrus by a visual inspection and an impedance value near or over 8 Kilo-Ohm.

The WT and *Thra1*^*PV/+*^ mice^15^ were induced to hyperthyroidism by treatment with 0.05% Methimazole (MMI) and 1% potassium perchlorate (KClO4) in the drinking water for 5 weeks. For induction of hyperthyroidism, mice were treated with an additional 0.5 µg/ml of T3 in drinking water in the 5th week. The control group was given normal drinking water.

### Body and uterus weight measurements

Female WT (*n* = 31) and *Thra1*^*PV/+*^ mice (*n* = 28), aged 2–3, 4–5, 6–7, and 7–12 months (7 to 8 mice per age group); average age for WT and *Thra1*^*PV/+*^ mice was 6.0 months and 5.3 months, respectively. Mice were weighed using an electronic scale. For euthanization protocol, per ACUC guidelines, mice were placed in a CO2 euthanasia chamber, with the gas flow rate gradually increased to 20–30% of the chamber volume per minute. For a standard sized mouse cage, the flow rate was set at 5.5 L per minute to induce unconsciousness. Death was confirmed by checking for the absence of heartbeat, respiration, and reflexes. After euthanasia, necropsy was performed at the Molecular Histopathology Lab in Frederick National Lab; tissues were evaluated grossly and fixed in 10% neutral buffered formalin (Sigma, St. Louis, MO). Carcasses were disposed of according to institutional protocols.

### Histological analysis

The female reproductive tracts were trimmed to include the vagina, cervix, both uterine horns, and ovary from female WT and *Thra1*^*PV/+*^ mutant mice and embedded in paraffin. The slides were stained with H&E or Masson’s trichrome. The stained images were captured with a light microscope (Olympus LC 30 camera). For immunohistochemical analysis, the unstained slides were treated with antibodies for Ki-67, cleaved caspase-3, CD3, CD8a, Iba, CD11b, and antibodies as described (Kim et al., Thyroid, 2023). The sources and catalog numbers of the antibodies used are listed in the Supplementary Table 1.

### Western blot analysis

Tissue protein extraction reagent buffer (Thermo Fisher Scientific, Waltham, MA, USA) and phenylmethylsulfonyl fluoride (PMSF) were added to the dissected uterus, and the supernatant was collected by centrifugation at 13,000 rpm for 10 min. After protein quantification, lysates were heated at 100 °C for 5 min. Proteins were loaded on Tris-glycine gels (Thermo Fisher Scientific, Waltham, MA, USA) and transferred to 0.45 μm nitrocellulose membranes (Millipore, Burlington, MA). They were then incubated with antibodies against collagens and KLF9 overnight at 4 °C. Membranes were washed with TBS-T and incubated with secondary antibodies for 1 h at room temperature. After washing with TBS-T, proteins were detected using the ECL system (Enzo Life Sciences, New York, USA). Band intensities were quantified with ImageJ software (ImageJ 1.48v; Wayne Rasband, NIH). The antibodies used in the western blot analysis are listed in Supplementary Table 3.

### Gene expression by real-time quantitative and RNAScope analysis

Total RNA was extracted from tissues using TRIzol reagent (Thermo Fisher Scientific, Waltham, MA, USA), and RNA was purified by using PureLink™ (Thermo Fisher Scientific, Waltham, MA, USA). cDNA was synthesized using the GoScript™ Reverse Transcriptase Kit (Promega, Wisconsin, USA). Quantitative RT-PCR (qRT-PCR) was performed using the PowerUp™ SYBR™ Green Master Mix for qPCR Kit (Thermo Fishjer Scientific, Waltham, MA, USA). Primers used for qRT-PCR are listed in Supplementary Table 4.

The expression of *Il-33* and *Klf9* genes was also analyzed by RNAscope analysis. The expression of the *Il-33* and *Klf9* genes were detected by staining 5 μm FFPE mouse uteri sections with the RNAScope^®^ 2.5 LS probe Mm-IL-33 (ACD, Cat# 400598) or RNAScope^®^ 2.5 LS probe Mm-Klf9-C2 (ACD, Cat# 488378-C2) using the RNAscope LS Multiplex Fluorescent Assay (ACD, Cat# 322800). Using Bond RX auto-stainer (Leica Biosystems), the tissue was pre-treated 15 min at 95 °C with Bond Epitope Retrieval Solution 2 (Leica Biosystems), 15 min of Protease III (ACD) at 40 °C, and 1:750 dilution of OPAL™ 690 reagent (AKOYA, Biosciences^®^). The RNAscope 2.5 LS Negative Control Probe (Bacillus subtilis dihydrodipicolinate reductase (*dapB*) gene, cat# 312038) was used as a negative control. The RNAscope^®^ LS 2.5 Positive Control Probe Mm-PPIB (cat# 313918) was used as a technical control to ensure the RNA quality of tissue sections was suitable for staining. The *Il-33* and *Klf9* stained slides were digitally imaged using an Aperio FL scanner. Klf9 slides were digitally imaged using an PhenoImager^®^ HT 2.0 (AKOYA, Biosciences^®^).

### Laser-capture micro-dissected (LCM) sample preparation and RNA extraction for bulk RNA-sequencing

Serial, 10 μm thick, frozen sections of mouse uteri were mounted on PEN Membrane slides (ThermoFisher Scientific, cat# LCM0522). LCM slides were fixed in 3% glacial acetic acid/100% ethanol, stained 30 s. with Methyl green (Vector, cat# H3402) containing ProtectRNA (Sigma, cat# R7397) followed by 30 s. with 0.1% Cresyl violet acetate/0.1% Eosin Y (Epredia, cat# 7111)/50% ethanol, dehydrated in 100% alcohol, cleared in xylene, and air dried in the fume hood for 5 min and 15 min in a desiccator as previously described^50^. The endometrium was dissected from dried sections using an Arcturus XT laser capture microdissection instrument (ThermoFisher Scientific, U.S.A.). RNA was isolated by Qiagen RNeasy Micro extraction kit with the protocol for simultaneous recovery of small and large RNAs. RNA integrity and quantity was evaluated by Agilent Bioanalyzer PicoChip. Purified RNA was stored at -80 °C prior to RNA sequencing (RNA-seq and bioinformatic analysis is shown in Supplementary Information).

#### Digital spatial profiling sample processing, region of interest selection, and sequencing

Representative FFPE blocks for each age and genotype group were used to construct tissue microarrays (TMAs) using a Beecher MTA-1 tissue arrayer (Estigen Tissue Science), which included the endometrial cores from 24 WT mice and 24 *Thra1*^*PV/+*^ mice. For the NanoString GeoMx DSP RNA assays, slides were prepared using the Leica Biosystems BOND RX FFPE RNA Slide Preparation Protocol in the GeoMx NGS Slide Preparation User Manual (NanoString, MAN-10 115-04), and slides were processed for digital spatial profiling (DSP) within 5 days of microtomy. Slides were hybridized with the GeoMx Whole Transcriptome Atlas Mouse RNA (GMX-RNA-NGSMsWTA) and immunofluorescently labeled with anti-cytokeratin and anti-smooth muscle actin as morphology markers. The detailed methods and bioinformatic analysis are included in the Supplemental Information Section.

#### Statistical analysis

All statistical analyses and graphs were performed using GraphPad Prism v9.3 (GraphPad software). Statistical significance was determined using the two-tailed Student unpaired *t*-test. P-values < 0.05 were considered statistically significant. All data are expressed as the mean ± standard error of the mean.

## Electronic supplementary material

Below is the link to the electronic supplementary material.

Supplementary Material 1

Supplementary Material 2

Supplementary Material 3

## Data Availability

There are two GEO accession numbers:1). For bulk RNA-seq: The datasets generated and/or analyzed during the current study are available in the GEO with the accession number: GSE272299), which can be accessed by reviewers from the NCBI GEO website: https://www.ncbi.nlm.nih.gov/geo/query/acc.cgi? acc=GSE272299 2). Another is for GeoMx-DSP dataset: The GEO submission is GSE273927 and can be found here: https://www.ncbi.nlm.nih.gov/geo/query/acc.cgi? acc=GSE273927.
