# Supplementary material for: TRα1 mutant suppresses KLF9 to cause endometrial metaplasia with ectopic IL-33 expression leading to uterine fibrosis and infertility
Source: Sci Rep. 2025 Jan 31;15:3892. doi: 10.1038/s41598-025-86848-5 (PMC11785771; doi:10.1038/s41598-025-86848-5)
Supplement: Supplementary file 1 — Supplementary Material 1 [file 41598_2025_86848_MOESM1_ESM.docx]

**Supplementary figures and legends**

**TRα1 mutant suppresses KLF9 to cause endometrial metaplasia with ectopic IL-33 expression, leading to uterine fibrosis and infertility**

Elijah Edmondson^2^, Takahito Kimura^1^, Eunmi Hwang^1^, Minjun Kim^1^, Andrew Warner^2^, Yuelin Zhu^3^, Li Zhao^1^, Yan-lin Yu^1^, Xuguang Zhu^1^, Maria, Hernandez^4^, Noemi Kedei^4^, and Sheue-yann Cheng^1^*

^(1)^Laboratory of Molecular Biology, ^(3)^Laboratory of Cancer Biology, Center for Cancer Research, ^(4)^Collaborative Protein Technology Resources, Office of Science and Technology Resources, National Cancer Institute, National Institute of Health, Bethesda, and ^(2)^Molecular Histopathology Laboratory, Frederick National Laboratory for Cancer Research, Frederick, MD, USA

**Supplementary Figure 1**

**
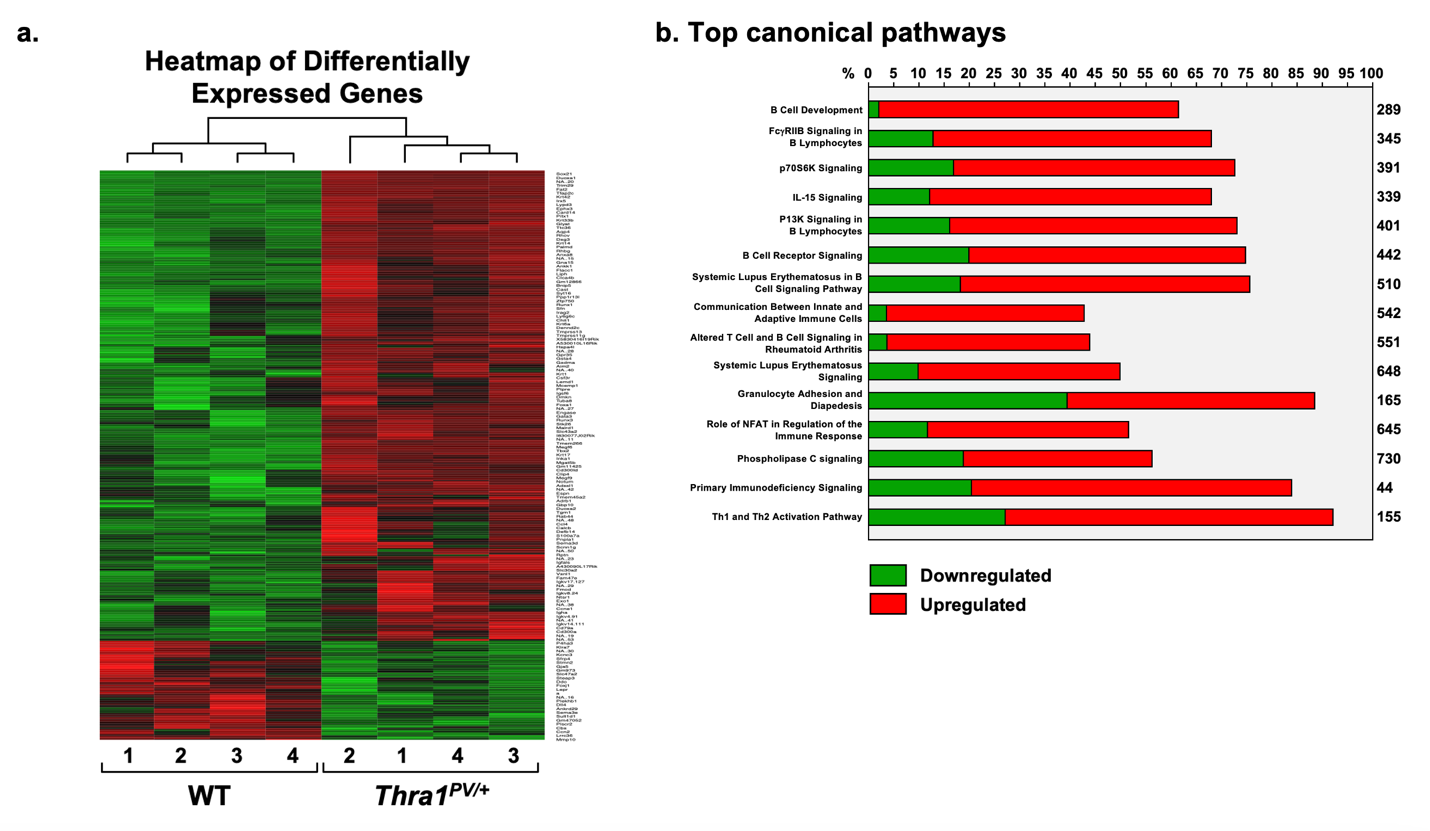
**

**Supplementary Figure 1. Distinct gene expression profiles between the laser-captured micro-dissected endometrium of WT and *Thra1^PV/+^* mice.** Female WT and *Thra1^PV/+^* mice were synced at the metestrus phase (n=4 for each of WT and *Thra1^PV/+^* mice). RNA-seq of laser-captured micro-dissected endometrium was analyzed as described in Methods. (a). **Heatmaps of differentially expressed genes**. The heatmap showed the differential expression in uterus tissues of the wild-type and *Thra1^PV/+^* mice. The differential genes were selected by fold changes >= 2 and adjusted p values <= 0.1. Of 592 genes differentially altered in the *Thra1^PV/+^* mice compared to the wild-type mice, 489 were up-regulated, and 102 were down-regulated. Heatmaps of differentially expressed genes. (b). The bar plot shows the top canonical pathways associated with the differential expression in uterus tissues by Ingenuity pathway analysis (IPA). The numbers in the Y axis are the total numbers of genes in a given pathway, and the bar lengths show the percentage of the differentially expressed genes that overlap with all the genes. The bars in green show the percentage of down-regulated genes, and the bars in red color show the percentage of up-regulated genes.

**
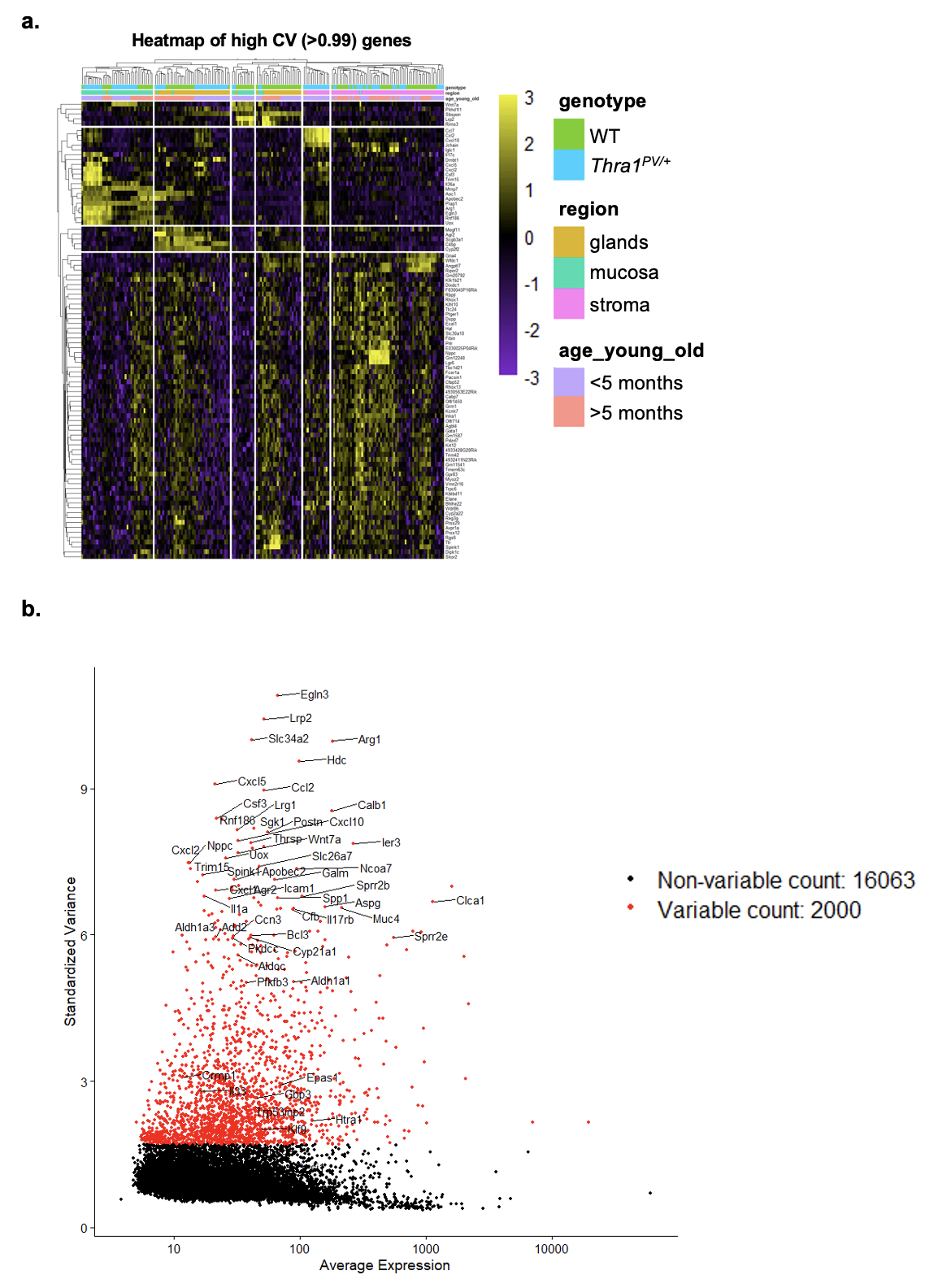
**

**Supplementary Figure 2. Unsupervised clustering of highly variable genes.**

**(a).**Unsupervised hierarchical clustering of 192 samples across using average clustering method. Samples cluster based on genotype, histologic region (endometrial mucosa, glands, and stroma), and age. **(b).**Detection of variable genes while controlling for the relationship between variability and average expression by calculating average expression and a z-score for dispersion.

**Supplementary Figure 3**

**
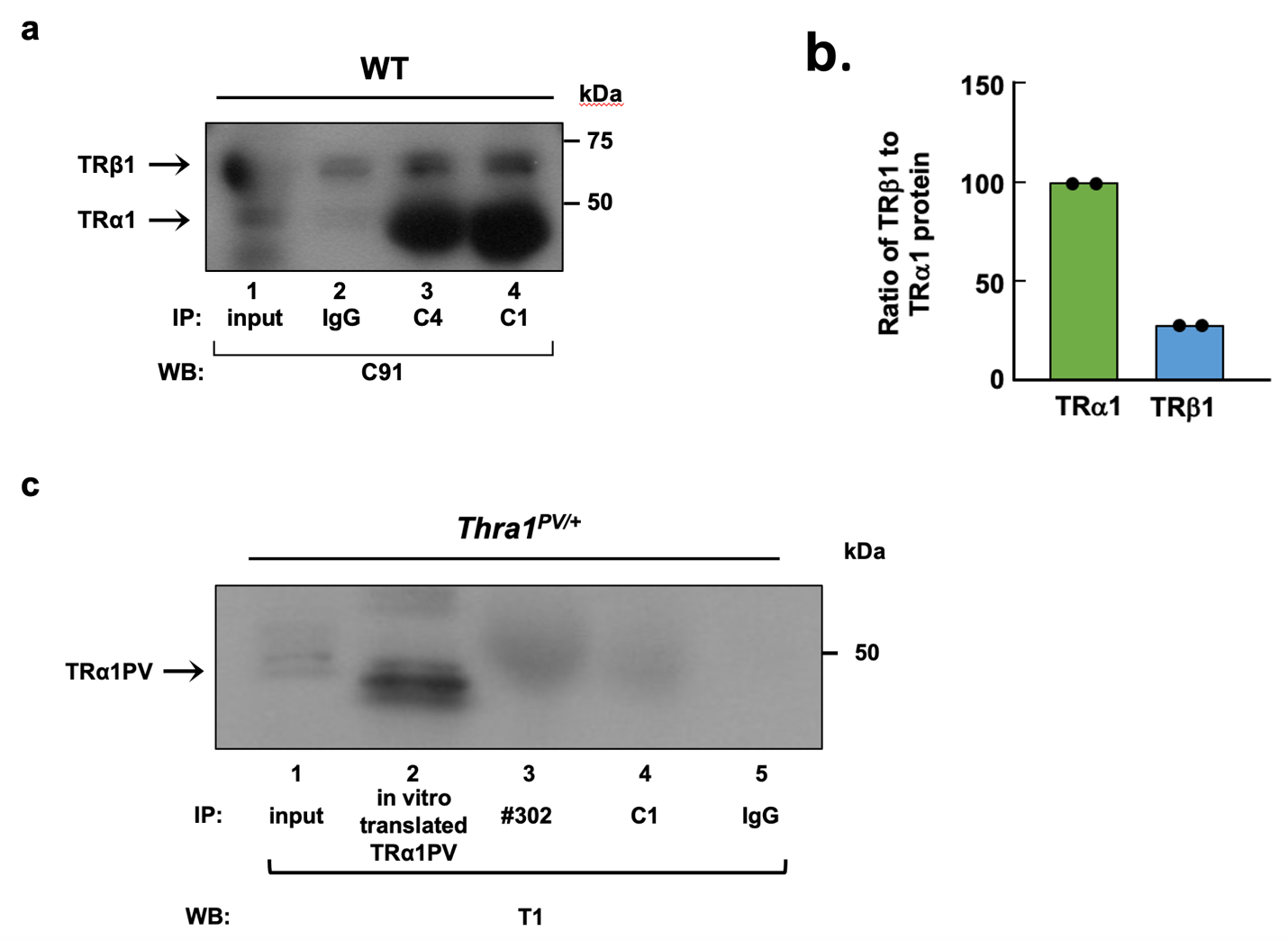
**

**Supplementary Figure 3. TRα1 is the major TR isoform in the mouse uterus.**

**(a).** Uterus extracts (500 µg) from WT mice months were first immunoprecipitated by rabbit anti-TR antibodies that recognized C-terminus of WT TRα1 and TRβ1 (C91, 4 µg/ml) followed by mouse monoclonal anti-TR antibodies C4 and C1 (2 µg/mL, each) that recognized both TR isoforms at the C-terminus and the region between DNA binding and ligand binding domains, respectively. The upper band with a higher molecular weight of ~56 kDa was TRβ1 and the lower band with molecular weight of ~48 kDa was TRα1. **(b).** The band intensities of TRβ1 and TRα1 were quantified and the determined ratio was: TRα1: TRα1=4.4:1. (**c).** Uterus extracts (500 µg) of *Thra1^PV/+^* mice were first immunoprecipitated by rabbit anti-TRα1PV specific antibodies, T1 (4 µg/ml), followed by monoclonal antibodies against TR, C1 (2 µg/ml; lane 4) and specifically recognized TRα1PV, #302 (2 µg/ml; lane 3), showing that TRα1PV was present in the uterus of *TRα1^PV/+^* mice. Antibody #302 specifically recognizes TRα1PV. Lane 1 was the input; lane 2: the TRα1PV proteins were prepared by using in vitro transcription and translation kit (TNT Quick Coupled Transcription/Translation System; Promega, Co)

**
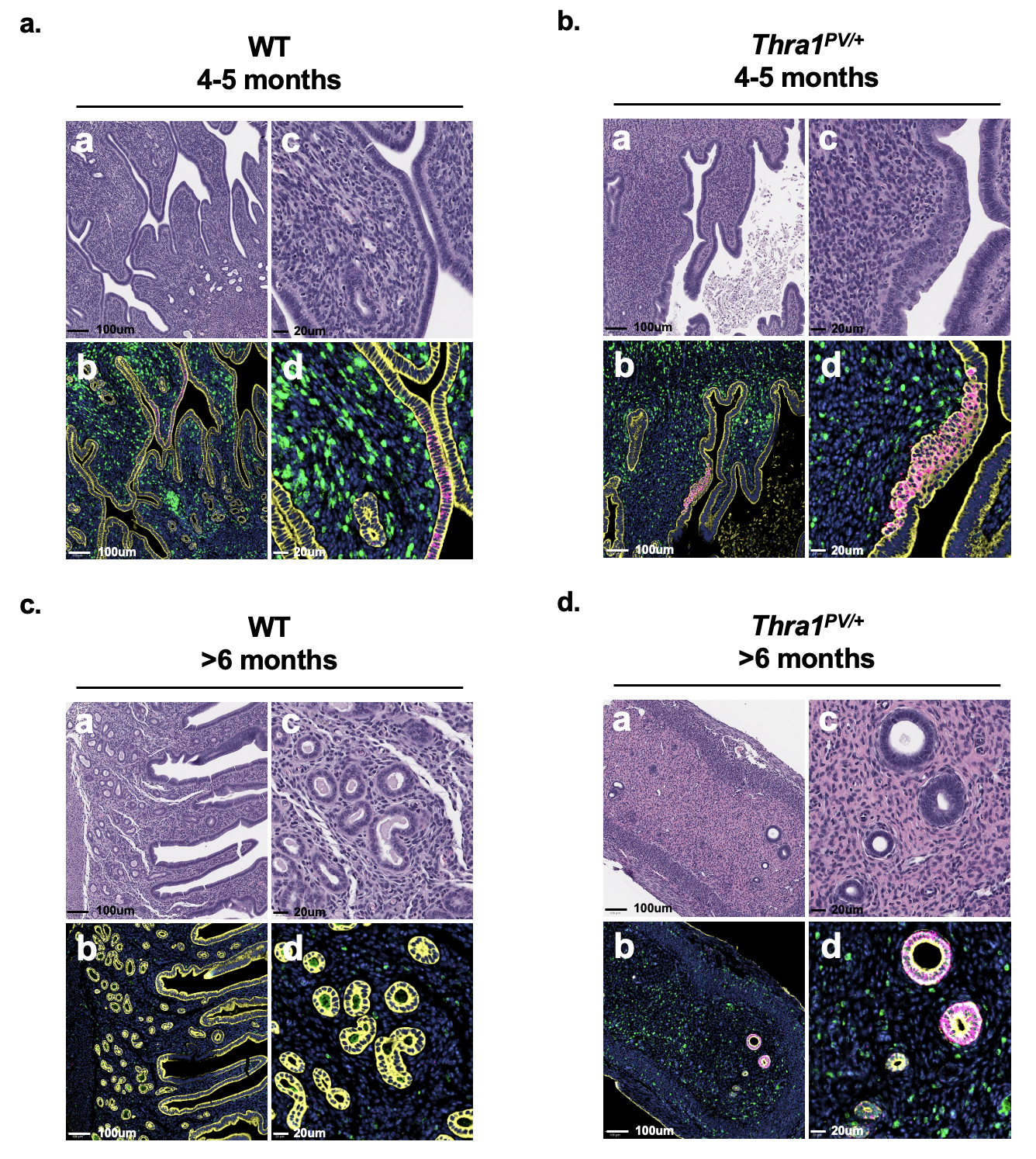
**

**Supplementary Figure 4. Association of endometrial squamous metaplasia with elevated expression of the *Il-33* gene in the epithelium of *Thra1^PV/+^* mice.**

*IL33* gene expression by RNAScope with cytokeratin (yellow) and CD45 (green) in the endometrium of WT mice (a); age: 4-5 months; c; >6 months) and *Thra1^PV/+^* mice (b), age: 4-5 months; d >6 months). *IL33* expression is rare in WT but common in *Thra1^PV/+^* endometrium. The source of IL-33 is epithelial cells. In *Thra1^PV/+^* mice, intense *Il-33* signal is corresponds to regions of endometrial squamous metaplasia. (Within the panels marked in white: Panel a and b, low magnification (bar=100 µm). Panel c and d, high magnification (bar=20 µm).

**Supplementary Figure 5**


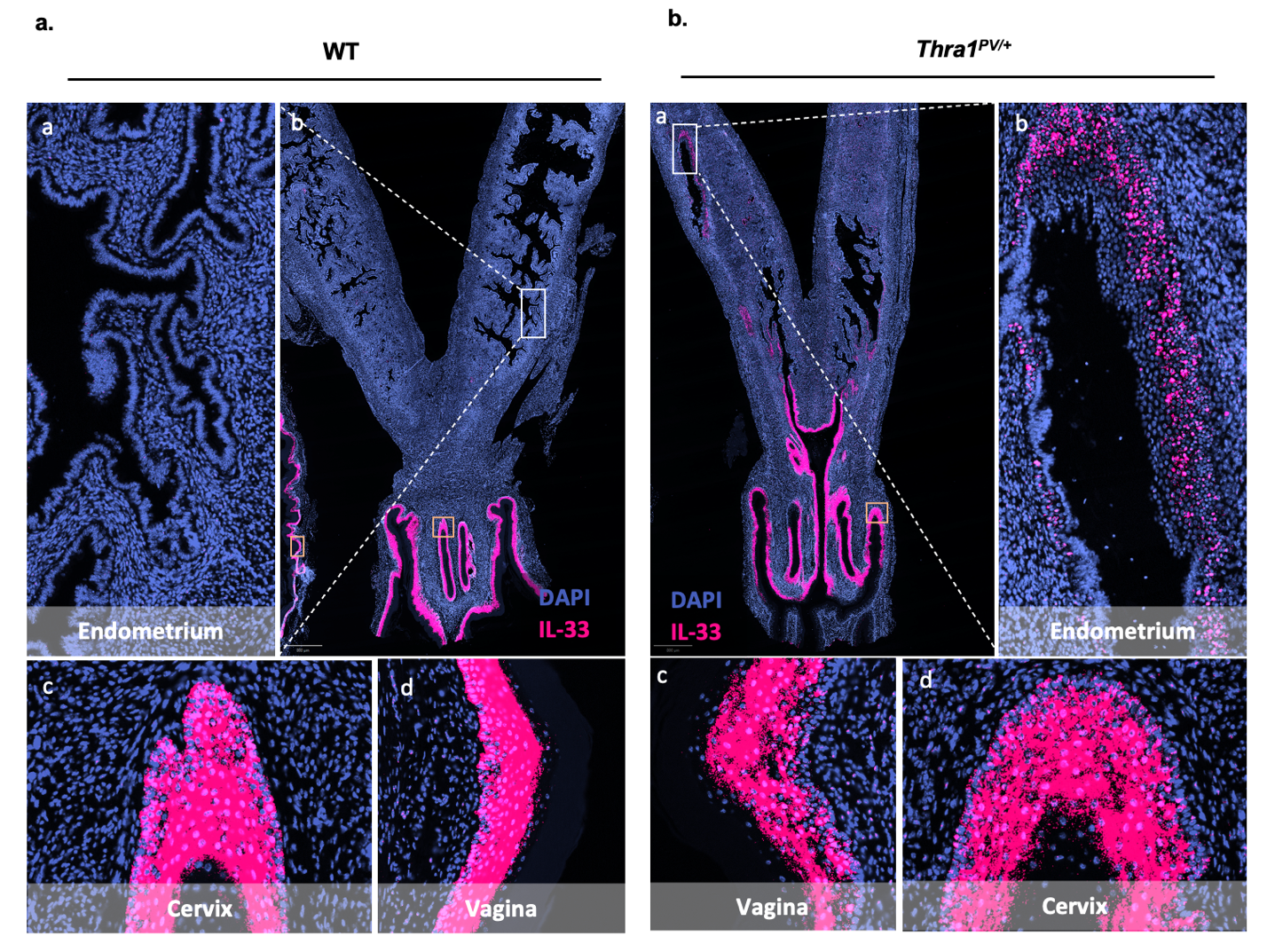


**Supplementary Figure 5. The expression of the *Il-33* gene expression in the female reproductive track of WT and *Thra1^PV/+^* mice. (a).** The expression of the *Il-33* gene is rare in the endometrium of WT mice; but was observed diffusely in the stratified squamous epithelium of the cervix and vagina. **(b).** The expression of the *Il-33* gene was detected in the endometrium as well as in the cervix and vagina of *Thra1^PV/+^* mice.

**Supplementary Figure 6**

**
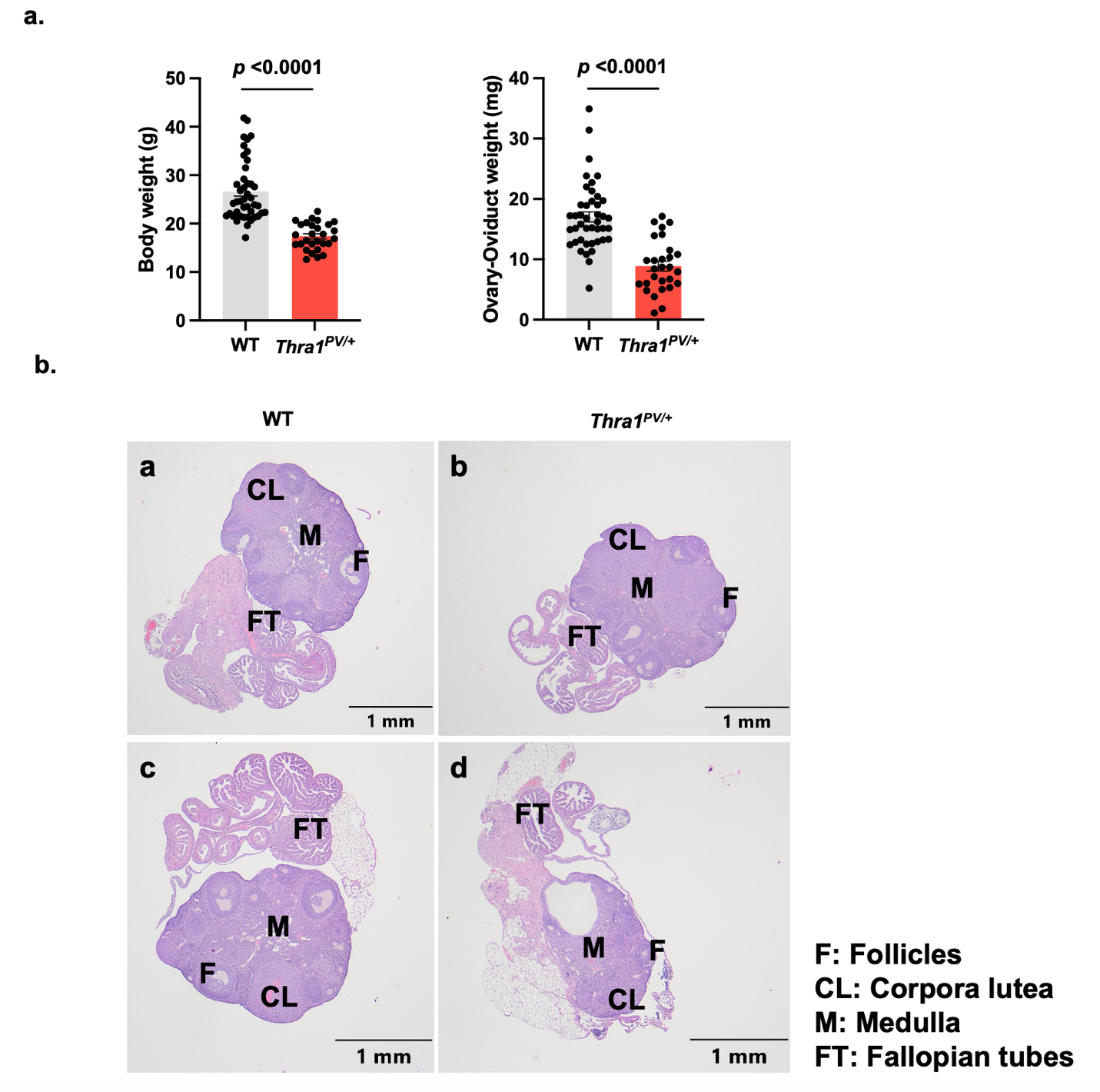
**

**Supplementary Figure 6. Histological analysis of ovary/oviduct of WT and *Thra1^PV/+^* mice.**

**(a).** Comparison of body weight and ovary/oviduct (b) of WT (n=32) and *Thra1^PV/+^* mice

**(**n=19) (age, 2.4 -11.8 months). **(b).** Histological micrographs of ovary and fallopian tubes. Ovary in WT and *Thra1^PV/+^* mice often have multiple follicles (F) and corpora lutea (CL). The highly vascular central medulla (M) and fallopian tubes (FT) are designated. No significant differences of these histological features were observed between WT and *Thra1^PV/+^* mice.

**Supplementary Figure 7**

**
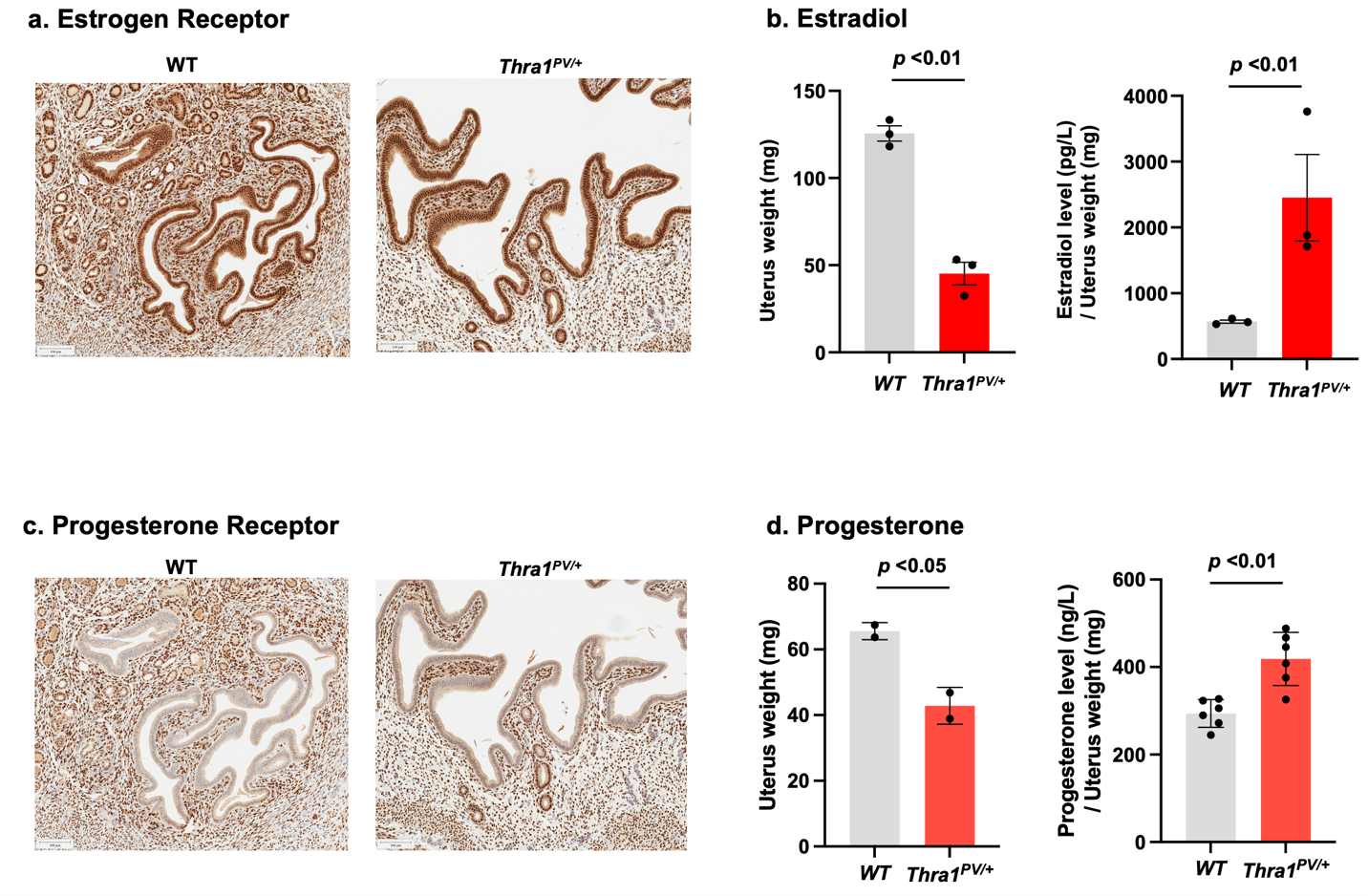
**

**Supplementary Figure 7 (a).** No apparent differences in the IHC staining of estrogen receptor in WT and mutant uterus. **(b)** Levels of estradiol were determined in the uterus of *WT* (n = 3) and *Thra1*^PV/+^ mice (n = 3). Decreased uterus weight in female *Thra1 ^PV/+^* mice (n = 3) compared to female WT mice (n = 3), mouse age: 2.9–5.4 months. An increased level of estradiol observed in *Thra1*^PV/+^ mice as compared with WT. The values were normalized to the weight of each uterus. Values are presented as means ± SEM. The *p*-values are indicated. **(c).** No apparent differences in the IHC staining of progesterone receptor in WT and mutant uterus. (**d**) Decreased uterus weight in female *Thra1 ^PV/+^* mice (n = 2) compared to female WT mice (n = 2), mouse age: 2.9–5.4 months. Progesterone levels were evaluated in the uterus of WT (n = 2) and *Thra1 ^PV/+^* mice (n = 2). An increased progesterone levels were observed in *Thra1 ^PV/+^* mice compared to WT mice. The values were normalized by the weight of the mouse uterus. Each sample was performed in triplicates. Values are presented as means ± SEM. The *p*-values are indicated.
