# Supplementary material for: TRα1 mutant suppresses KLF9 to cause endometrial metaplasia with ectopic IL-33 expression leading to uterine fibrosis and infertility
Source: Sci Rep. 2025 Jan 31;15:3892. doi: 10.1038/s41598-025-86848-5 (PMC11785771; doi:10.1038/s41598-025-86848-5)
Supplement: Supplementary file 2 — Supplementary Material 2 [file 41598_2025_86848_MOESM2_ESM.docx]

**Supplemental Information**

**TRα1 mutant suppresses KLF9 to cause endometrial metaplasia with ectopic IL-33 expression, leading to uterine fibrosis and infertility**

Elijah Edmondson^2^, Takahito Kimura^1^, Eunmi Hwang^1^, Minjun Kim^1^, Andrew Warner^2^, Yuelin Zhu^3^, Li Zhao^1^, Yan-lin Yu^1^, Xuguang Zhu^1^, Maria, Hernandez^4^, Noemi Kedei^4^, and Sheue-yann Cheng^1^*

^(1)^Laboratory of Molecular Biology, ^(3)^Laboratory of Cancer Biology, Center for Cancer Research, ^(4)^Collaborative Protein Technology Resources, Office of Science and Technology Resources, National Cancer Institute, National Institute of Health, Bethesda, and ^(2)^Molecular Histopathology Laboratory, Frederick National Laboratory for Cancer Research, Frederick, MD, USA

**Materials and Methods**

***Bulk RNA-seq analysis of laser-captured dissected endometrium of WT and Thra1 ^PV/+^ mice***

The libraries were prepared according to the Illumina Stranded mRNA Prep protocol for paired-end sequencing. The libraries were pooled and sequenced on an Illumina NextSeq 2000 sequencer. After confirmed good sequence quality, the raw reads of the samples were processed with common RNA-Seq processing procedure, including trimming reads for removing adapters and low-quality bases using Trimmomatic software. The transcript level raw counts were computed with the *salmon quant* method from Salmon software (1). DESeq2 Bioconductor package (2) was used for normalization, summarization and differential expression analysis. Mouse genome mm10 was used in the analysis. The differential genes were selected by fold changes >= 2 and adjusted p values <= 0.1.

***Additional information for digital spatial profiling sample processing, region of interest selection, and sequencing.***

For the NanoString GeoMx DSP RNA assays, slides were processed within 5 days after the microtomy for the NanoString GeoMx DSP RNA assay following the GeoMx NGS Slide Preparation User Manual (NanoString, MAN-10 115-04). After 30 min baking at 60^o^C, dewaxing, heat-induced epitope retrieval (ER2 for 20 min at 100^o^C) and proteinase K treatment (1 mg/mL proteinase K in 1X phosphate-buffered saline (PBS) at 37°C for 15 min) was performed on Leica Biosystems BOND RX according to the FFPE RNA Slide Preparation Protocol. Slides were hybridized with the GeoMx Whole Transcriptome Atlas Mouse RNA for Illumina Panel (GMX[1]RNA-NGSMsWTA) overnight at 37^o^C, followed by a series of stringent washes and morphology staining: Syto 13 for nuclei, pancytokeratin- AF532 to visualize epithelial cells and smooth muscle actin (aSMA-AF647) to visualize stroma. The staining was visualized on the GeoMx DSP instrument using a 20X objective on FITC, CY3, Texas Red and CY5 channels.  A total of 190 regions of interest (ROI) were selected from 13 mouse uterus sections (6 *Thra1 ^PV/+^* and 7 WT mice), which included endometrial mucosa, glands, and stroma; a minimum of 5 ROIs were taken per category per mouse. Annotations were selected by a board-certified veterinary pathologist based on morphology and marker staining using HALO (Indica Labs). ROI masks were imported to the GeoMx and used to guide UV light cleavage of the barcode linkers of the prebound RNA probes in each area of illumination, and the cleaved probes were deposited in DSP collection plates. Library construction and sequencing (NextSeq2000) was performed according to Nanostring’s Guidelines at the Genomics Core (OSTR, CCR). Processed data was uploaded to the DSP analysis suite for visualization and quality control while detailed data analysis was performed using an R-package from Nanostring.

*Bioinformatic Analysis, Quality Control and* *Gene Filtering of GeoMx DSP data.*

Raw data was evaluated in R (version 4.1.2). ROI segment and probe quality control (QC) was performed according to NanoString’s Whole Transcriptome Atlas Normalization guidelines. ROI QC was performed using the GeomxTools (V3.2.0) package. ROIs were flagged using the following settings: raw reads threshold 1000 reads, percent trimmed 80%, percent stitched 80%, percent aligned reads 80%, percent sequencing saturation 50%, negative probe count geomean 1, no template PCR control count > 60, minimum nuclei 20, and minimum surface area of 1000 µm². Of the 190 AOIs, one was flagged for a low negative probe count and low sequencing alignment. Probe QC was performed with default settings excluding probes from target count calculation in all segments if (geomean probe in all segments)/(geomean probes within target) ≤ 0.1 and if it fails Grubbs’ outlier test in ≥20% of the segments. The limit of quantification (LOQ) was calculated using two SDs of the geomean of the negative probes. Quartile 3 count (Q3) normalization was performed to account for technical effects between AOIs. Gene filtering was used to filter and select only genes detected in at least 10% of AOIs. Following QC, the resulting gene expression matrix consisted of 189 AOIs that passed QC and a total of 14,882 genes. Q3-normalised expression data was exported from the DSP Analysis Suite. Dimension-reduction of the expression matrix was performed using t-Distributed Stochastic Neighbor Embedding by R package Rtsne (V.0.16), umap (V.0.2.10.0), and visualized using ggplot2 (V.3.4.3).

*Differentially expression genes (DEGs) and Gene Set Enrichment Analyses.*

DEGs between and N/N mucosa, gland, and stroma segments were determined using a Linear Mixed Model GeoMxTools Likelihood ratio test, assuming an underlying negative binomial distribution within Seurat (V.5.0.1) (3). Single-sample gene set enrichment analysis (ssGSEA, from GSVA v1.41.3) was used to score transcriptional pathways for each AOI (4). Gene Ontology was retrieved using the downloadPathwayArchive function of the R package clusterProfiler AnnotationDbi::select(org.Mm.eg.db, keys=keys(org.Mm.eg.db), columns=GO) in February 2024 and pathway over-representation analysis (ORA) was performed (5).

**References**

1. R. Patro, G. Duggal, M. I. Love, R. A. Irizarry, C. Kingsford, Salmon provides fast and bias-aware quantification of transcript expression. *Nat Methods* **14**, 417-419 (2017).

2. M. I. Love, W. Huber, S. Anders, Moderated estimation of fold change and dispersion for RNA-seq data with DESeq2. *Genome Biol* **15**, 550 (2014).

3. Y. Hao *et al.*, Dictionary learning for integrative, multimodal and scalable single-cell analysis. *Nat Biotechnol* **42**, 293-304 (2024).

4. S. Hanzelmann, R. Castelo, J. Guinney, GSVA: gene set variation analysis for microarray and RNA-seq data. *BMC Bioinformatics* **14**, 7 (2013).

5. G. Yu, L. G. Wang, Y. Han, Q. Y. He, clusterProfiler: an R package for comparing biological themes among gene clusters. *OMICS* **16**, 284-287 (2012).

**Supplementary Table 1. List of Differential expression genes is listed separately**

| Sbspon | 1.46E-07 | 1.357 | 2.63E-03 | FDR < 0.05 | 6.84 |
| --- | --- | --- | --- | --- | --- |
| Pi15 | 1.42E-07 | 0.861 | 2.56E-03 | FDR < 0.05 | 6.85 |
| Nptx1 | 1.37E-07 | 0.695 | 2.47E-03 | FDR < 0.05 | 6.86 |
| Cavin3 | 1.36E-07 | 1.242 | 2.46E-03 | FDR < 0.05 | 6.87 |
| Spns2 | 1.35E-07 | 0.945 | 2.45E-03 | FDR < 0.05 | 6.87 |
| Il31ra | 1.34E-07 | 0.605 | 2.42E-03 | FDR < 0.05 | 6.87 |
| Clcn2 | 1.34E-07 | 0.575 | 2.42E-03 | FDR < 0.05 | 6.87 |
| Lamc3 | 1.32E-07 | 0.633 | 2.39E-03 | FDR < 0.05 | 6.88 |
| Rasgrp3 | 1.22E-07 | 0.641 | 2.21E-03 | FDR < 0.05 | 6.91 |
| Nat8f3 | 1.21E-07 | 0.625 | 2.18E-03 | FDR < 0.05 | 6.92 |
| Dock8 | 1.19E-07 | 0.567 | 2.14E-03 | FDR < 0.05 | 6.93 |
| Adamts15 | 1.18E-07 | 0.594 | 2.14E-03 | FDR < 0.05 | 6.93 |
| Htra3 | 1.18E-07 | 0.749 | 2.12E-03 | FDR < 0.05 | 6.93 |
| Dcdc2b | 1.06E-07 | 0.586 | 1.91E-03 | FDR < 0.05 | 6.98 |
| Medag | 1.03E-07 | 0.633 | 1.86E-03 | FDR < 0.05 | 6.99 |
| Tmtc1 | 9.48E-08 | 0.818 | 1.71E-03 | FDR < 0.05 | 7.02 |
| Adamts1 | 9.25E-08 | 0.581 | 1.67E-03 | FDR < 0.05 | 7.03 |
| Abhd1 | 9.21E-08 | 0.539 | 1.66E-03 | FDR < 0.05 | 7.04 |
| Magix | 9.06E-08 | 0.662 | 1.64E-03 | FDR < 0.05 | 7.04 |
| Asl | 9.03E-08 | 0.507 | 1.63E-03 | FDR < 0.05 | 7.04 |
| Secisbp2l | 8.89E-08 | 0.536 | 1.61E-03 | FDR < 0.05 | 7.05 |
| Myct1 | 8.88E-08 | 0.634 | 1.60E-03 | FDR < 0.05 | 7.05 |
| Aurkc | 8.80E-08 | 0.628 | 1.59E-03 | FDR < 0.05 | 7.06 |
| Krtap28-13 | 8.50E-08 | 0.614 | 1.53E-03 | FDR < 0.05 | 7.07 |
| Ahsg | 8.40E-08 | 1.336 | 1.52E-03 | FDR < 0.05 | 7.08 |
| Il16 | 7.64E-08 | 0.59 | 1.38E-03 | FDR < 0.05 | 7.12 |
| Gm6358 | 7.16E-08 | 0.627 | 1.29E-03 | FDR < 0.05 | 7.14 |
| Phldb2 | 6.88E-08 | 0.541 | 1.24E-03 | FDR < 0.05 | 7.16 |
| Hilpda | 6.01E-08 | 0.68 | 1.09E-03 | FDR < 0.05 | 7.22 |
| Dppa5a | 5.96E-08 | 0.653 | 1.08E-03 | FDR < 0.05 | 7.22 |
| Dab2 | 5.65E-08 | 0.677 | 1.02E-03 | FDR < 0.05 | 7.25 |
| Dab1 | 5.60E-08 | 1.137 | 1.01E-03 | FDR < 0.05 | 7.25 |
| Gm52520 | 4.86E-08 | 0.705 | 8.78E-04 | FDR < 0.001 | 7.31 |
| Adipor2 | 4.83E-08 | 0.587 | 8.72E-04 | FDR < 0.001 | 7.32 |
| Ric1 | 4.82E-08 | 0.551 | 8.71E-04 | FDR < 0.001 | 7.32 |
| Kcnma1 | 4.81E-08 | 0.792 | 8.69E-04 | FDR < 0.001 | 7.32 |
| Tcf23 | 4.77E-08 | 0.79 | 8.62E-04 | FDR < 0.001 | 7.32 |
| Duxf3 | 4.70E-08 | 0.786 | 8.50E-04 | FDR < 0.001 | 7.33 |
| Tmem117 | 4.57E-08 | 0.57 | 8.26E-04 | FDR < 0.001 | 7.34 |
| Wnt5a | 4.54E-08 | 0.523 | 8.19E-04 | FDR < 0.001 | 7.34 |
| Mid1ip1 | 4.52E-08 | 0.62 | 8.17E-04 | FDR < 0.001 | 7.34 |
| Adam12 | 4.50E-08 | 0.669 | 8.13E-04 | FDR < 0.001 | 7.35 |
| Ippk | 4.39E-08 | 0.788 | 7.92E-04 | FDR < 0.001 | 7.36 |
| Thy1 | 4.32E-08 | 0.988 | 7.80E-04 | FDR < 0.001 | 7.36 |
| Vmn2r46 | 4.31E-08 | 0.667 | 7.78E-04 | FDR < 0.001 | 7.37 |
| LOC118568634 | 4.21E-08 | 0.63 | 7.61E-04 | FDR < 0.001 | 7.38 |
| Smarca2 | 3.93E-08 | 0.598 | 7.10E-04 | FDR < 0.001 | 7.41 |
| Has2 | 3.88E-08 | 0.586 | 7.00E-04 | FDR < 0.001 | 7.41 |
| Lbp | 3.73E-08 | 0.972 | 6.73E-04 | FDR < 0.001 | 7.43 |
| Vit | 3.69E-08 | 0.763 | 6.67E-04 | FDR < 0.001 | 7.43 |
| Slc51a | 3.36E-08 | 0.869 | 6.07E-04 | FDR < 0.001 | 7.47 |
| Socs7 | 3.32E-08 | 0.597 | 5.99E-04 | FDR < 0.001 | 7.48 |
| Gli1 | 3.23E-08 | 1.025 | 5.84E-04 | FDR < 0.001 | 7.49 |
| Glce | 2.96E-08 | 0.593 | 5.34E-04 | FDR < 0.001 | 7.53 |
| Rem1 | 2.82E-08 | 0.529 | 5.10E-04 | FDR < 0.001 | 7.55 |
| Cd302 | 2.81E-08 | 0.577 | 5.08E-04 | FDR < 0.001 | 7.55 |
| Itprid2 | 2.74E-08 | 0.594 | 4.95E-04 | FDR < 0.001 | 7.56 |
| Mfhas1 | 2.52E-08 | 0.506 | 4.55E-04 | FDR < 0.001 | 7.60 |
| Rcn1 | 2.49E-08 | 1.017 | 4.49E-04 | FDR < 0.001 | 7.60 |
| Gata2 | 2.37E-08 | 0.748 | 4.28E-04 | FDR < 0.001 | 7.63 |
| D830030K20Rik | 2.35E-08 | 0.641 | 4.25E-04 | FDR < 0.001 | 7.63 |
| Asb1 | 2.34E-08 | 0.553 | 4.23E-04 | FDR < 0.001 | 7.63 |
| D430041D05Rik | 2.21E-08 | 0.62 | 4.00E-04 | FDR < 0.001 | 7.65 |
| Adamts16 | 2.17E-08 | 1.083 | 3.93E-04 | FDR < 0.001 | 7.66 |
| Gm16434 | 2.07E-08 | 0.723 | 3.74E-04 | FDR < 0.001 | 7.68 |
| Rgs2 | 2.02E-08 | 0.825 | 3.66E-04 | FDR < 0.001 | 7.69 |
| Gm34826 | 1.96E-08 | 0.72 | 3.54E-04 | FDR < 0.001 | 7.71 |
| Pde4c | 1.91E-08 | 0.645 | 3.45E-04 | FDR < 0.001 | 7.72 |
| Pced1b | 1.88E-08 | 0.578 | 3.40E-04 | FDR < 0.001 | 7.72 |
| Rtn1 | 1.87E-08 | 0.963 | 3.38E-04 | FDR < 0.001 | 7.73 |
| Sult5a1 | 1.76E-08 | 0.808 | 3.18E-04 | FDR < 0.001 | 7.75 |
| Aldh6a1 | 1.75E-08 | 0.561 | 3.16E-04 | FDR < 0.001 | 7.76 |
| Herc1 | 1.74E-08 | 0.57 | 3.15E-04 | FDR < 0.001 | 7.76 |
| LOC118568783 | 1.74E-08 | 0.566 | 3.14E-04 | FDR < 0.001 | 7.76 |
| Osr2 | 1.65E-08 | 0.87 | 2.99E-04 | FDR < 0.001 | 7.78 |
| A130010J15Rik | 1.64E-08 | 0.527 | 2.97E-04 | FDR < 0.001 | 7.78 |
| E230025N22Rik | 1.60E-08 | 0.532 | 2.88E-04 | FDR < 0.001 | 7.80 |
| Cdo1 | 1.55E-08 | 0.913 | 2.80E-04 | FDR < 0.001 | 7.81 |
| Basp1 | 1.47E-08 | 0.684 | 2.65E-04 | FDR < 0.001 | 7.83 |
| Borcs5 | 1.43E-08 | 0.522 | 2.59E-04 | FDR < 0.001 | 7.84 |
| Tmem100 | 1.41E-08 | 0.708 | 2.55E-04 | FDR < 0.001 | 7.85 |
| Fam83a | 1.35E-08 | 1.244 | 2.45E-04 | FDR < 0.001 | 7.87 |
| Vmn1r113 | 1.33E-08 | 0.721 | 2.41E-04 | FDR < 0.001 | 7.87 |
| Numbl | 1.21E-08 | 0.706 | 2.18E-04 | FDR < 0.001 | 7.92 |
| Sesn1 | 1.12E-08 | 0.525 | 2.03E-04 | FDR < 0.001 | 7.95 |
| Dzank1 | 1.12E-08 | 0.575 | 2.02E-04 | FDR < 0.001 | 7.95 |
| Ppp2r2c | 1.10E-08 | 1.148 | 1.99E-04 | FDR < 0.001 | 7.96 |
| Dgkg | 1.09E-08 | 0.983 | 1.96E-04 | FDR < 0.001 | 7.96 |
| Aqp11 | 1.06E-08 | 1.022 | 1.91E-04 | FDR < 0.001 | 7.98 |
| Arg2 | 1.03E-08 | 1.017 | 1.87E-04 | FDR < 0.001 | 7.99 |
| Kcnc4 | 9.66E-09 | 0.808 | 1.74E-04 | FDR < 0.001 | 8.02 |
| Synpo2 | 8.90E-09 | 0.775 | 1.61E-04 | FDR < 0.001 | 8.05 |
| Faiml | 8.61E-09 | 0.7 | 1.56E-04 | FDR < 0.001 | 8.07 |
| Gpc3 | 8.57E-09 | 0.865 | 1.55E-04 | FDR < 0.001 | 8.07 |
| Efhd1 | 8.13E-09 | 0.585 | 1.47E-04 | FDR < 0.001 | 8.09 |
| Arhgap24 | 7.82E-09 | 0.763 | 1.41E-04 | FDR < 0.001 | 8.11 |
| Lvrn | 7.72E-09 | 1.251 | 1.39E-04 | FDR < 0.001 | 8.11 |
| Arhgef10 | 6.82E-09 | 0.598 | 1.23E-04 | FDR < 0.001 | 8.17 |
| Hoxa10 | 5.95E-09 | 1.163 | 1.07E-04 | FDR < 0.001 | 8.23 |
| Znhit6 | 5.40E-09 | 0.966 | 9.75E-05 | FDR < 0.001 | 8.27 |
| D430019H16Rik | 5.34E-09 | 0.634 | 9.64E-05 | FDR < 0.001 | 8.27 |
| Fgf1 | 4.58E-09 | 1.038 | 8.27E-05 | FDR < 0.001 | 8.34 |
| Gm3248 | 4.50E-09 | 0.725 | 8.12E-05 | FDR < 0.001 | 8.35 |
| Btbd35f1 | 3.64E-09 | 0.731 | 6.57E-05 | FDR < 0.001 | 8.44 |
| Gm10662 | 3.47E-09 | 0.739 | 6.26E-05 | FDR < 0.001 | 8.46 |
| Pcx | 3.31E-09 | 1.253 | 5.98E-05 | FDR < 0.001 | 8.48 |
| Lama2 | 3.28E-09 | 0.859 | 5.92E-05 | FDR < 0.001 | 8.48 |
| Il15 | 2.83E-09 | 0.543 | 5.11E-05 | FDR < 0.001 | 8.55 |
| Golga5 | 2.72E-09 | 0.549 | 4.92E-05 | FDR < 0.001 | 8.56 |
| Gm2237 | 2.69E-09 | 0.685 | 4.85E-05 | FDR < 0.001 | 8.57 |
| Rhoj | 2.62E-09 | 0.649 | 4.72E-05 | FDR < 0.001 | 8.58 |
| Gm3383 | 2.52E-09 | 0.654 | 4.55E-05 | FDR < 0.001 | 8.60 |
| ccdc198 | 2.34E-09 | 0.976 | 4.22E-05 | FDR < 0.001 | 8.63 |
| Inmt | 2.05E-09 | 0.986 | 3.70E-05 | FDR < 0.001 | 8.69 |
| Krt23 | 2.04E-09 | 1.548 | 3.68E-05 | FDR < 0.001 | 8.69 |
| Ugp2 | 1.88E-09 | 0.564 | 3.40E-05 | FDR < 0.001 | 8.72 |
| Rab3a | 1.73E-09 | 0.56 | 3.13E-05 | FDR < 0.001 | 8.76 |
| Grin2b | 1.71E-09 | 1.262 | 3.09E-05 | FDR < 0.001 | 8.77 |
| Slit3 | 1.62E-09 | 0.851 | 2.93E-05 | FDR < 0.001 | 8.79 |
| Pkdcc | 1.43E-09 | 1.127 | 2.58E-05 | FDR < 0.001 | 8.85 |
| Chdh | 1.39E-09 | 0.712 | 2.50E-05 | FDR < 0.001 | 8.86 |
| Vmn1r100 | 1.31E-09 | 0.7 | 2.36E-05 | FDR < 0.001 | 8.88 |
| LOC118568632 | 1.26E-09 | 0.716 | 2.28E-05 | FDR < 0.001 | 8.90 |
| Fam234b | 1.22E-09 | 0.618 | 2.20E-05 | FDR < 0.001 | 8.92 |
| Hoxa11 | 1.19E-09 | 1.142 | 2.16E-05 | FDR < 0.001 | 8.92 |
| Ccn2 | 1.19E-09 | 1.598 | 2.16E-05 | FDR < 0.001 | 8.92 |
| Chrd | 1.15E-09 | 0.868 | 2.07E-05 | FDR < 0.001 | 8.94 |
| Kcnd2 | 1.11E-09 | 0.909 | 2.01E-05 | FDR < 0.001 | 8.95 |
| D630003M21Rik | 1.09E-09 | 0.692 | 1.97E-05 | FDR < 0.001 | 8.96 |
| Tmem52 | 1.08E-09 | 0.611 | 1.95E-05 | FDR < 0.001 | 8.97 |
| LOC118567641 | 9.80E-10 | 0.813 | 1.77E-05 | FDR < 0.001 | 9.01 |
| Jazf1 | 8.35E-10 | 0.813 | 1.51E-05 | FDR < 0.001 | 9.08 |
| Gm3696 | 8.12E-10 | 0.647 | 1.47E-05 | FDR < 0.001 | 9.09 |
| Galnt18 | 7.57E-10 | 0.718 | 1.37E-05 | FDR < 0.001 | 9.12 |
| Matn2 | 7.41E-10 | 0.798 | 1.34E-05 | FDR < 0.001 | 9.13 |
| Suox | 6.58E-10 | 0.694 | 1.19E-05 | FDR < 0.001 | 9.18 |
| Cnn3 | 6.50E-10 | 1.031 | 1.17E-05 | FDR < 0.001 | 9.19 |
| Mt3 | 6.29E-10 | 0.897 | 1.14E-05 | FDR < 0.001 | 9.20 |
| Ltbp4 | 6.15E-10 | 1.073 | 1.11E-05 | FDR < 0.001 | 9.21 |
| Slc13a5 | 6.12E-10 | 1.228 | 1.11E-05 | FDR < 0.001 | 9.21 |
| Gpt2 | 5.56E-10 | 0.678 | 1.00E-05 | FDR < 0.001 | 9.26 |
| Gm52436 | 5.49E-10 | 0.803 | 9.92E-06 | FDR < 0.001 | 9.26 |
| Faim2 | 4.77E-10 | 0.828 | 8.62E-06 | FDR < 0.001 | 9.32 |
| Naa80 | 4.71E-10 | 0.769 | 8.51E-06 | FDR < 0.001 | 9.33 |
| Fst | 3.18E-10 | 1.133 | 5.75E-06 | FDR < 0.001 | 9.50 |
| Aqp1 | 3.05E-10 | 0.826 | 5.51E-06 | FDR < 0.001 | 9.52 |
| Gm51591 | 2.87E-10 | 0.695 | 5.19E-06 | FDR < 0.001 | 9.54 |
| Fbn1 | 2.67E-10 | 0.968 | 4.82E-06 | FDR < 0.001 | 9.57 |
| Vldlr | 2.38E-10 | 0.736 | 4.30E-06 | FDR < 0.001 | 9.62 |
| Gm31035 | 2.31E-10 | 0.844 | 4.17E-06 | FDR < 0.001 | 9.64 |
| Cxcl12 | 2.31E-10 | 1.082 | 4.17E-06 | FDR < 0.001 | 9.64 |
| Etnk1 | 2.25E-10 | 1.002 | 4.07E-06 | FDR < 0.001 | 9.65 |
| Cdc14a | 1.92E-10 | 0.71 | 3.47E-06 | FDR < 0.001 | 9.72 |
| Gm9639 | 1.89E-10 | 0.714 | 3.42E-06 | FDR < 0.001 | 9.72 |
| Pknox2 | 1.87E-10 | 1.094 | 3.38E-06 | FDR < 0.001 | 9.73 |
| Lrrc3b | 1.78E-10 | 0.956 | 3.22E-06 | FDR < 0.001 | 9.75 |
| 4931406C07Rik | 1.74E-10 | 0.565 | 3.15E-06 | FDR < 0.001 | 9.76 |
| Bbx | 1.71E-10 | 0.553 | 3.09E-06 | FDR < 0.001 | 9.77 |
| Heg1 | 1.59E-10 | 0.722 | 2.87E-06 | FDR < 0.001 | 9.80 |
| Gm52800 | 1.55E-10 | 0.602 | 2.81E-06 | FDR < 0.001 | 9.81 |
| Pof1b | 1.42E-10 | 0.783 | 2.57E-06 | FDR < 0.001 | 9.85 |
| Nrn1 | 1.40E-10 | 1.015 | 2.53E-06 | FDR < 0.001 | 9.85 |
| Ramp1 | 1.38E-10 | 0.76 | 2.50E-06 | FDR < 0.001 | 9.86 |
| Cln5 | 1.31E-10 | 1.247 | 2.36E-06 | FDR < 0.001 | 9.88 |
| Tmprss2 | 1.30E-10 | 0.547 | 2.35E-06 | FDR < 0.001 | 9.89 |
| Ctsd | 1.29E-10 | 1.687 | 2.34E-06 | FDR < 0.001 | 9.89 |
| Kbtbd8 | 1.12E-10 | 0.915 | 2.02E-06 | FDR < 0.001 | 9.95 |
| Adcy9 | 1.05E-10 | 0.745 | 1.90E-06 | FDR < 0.001 | 9.98 |
| Lcmt1 | 9.89E-11 | 0.573 | 1.79E-06 | FDR < 0.001 | 10.00 |
| Nudt19 | 9.81E-11 | 0.863 | 1.77E-06 | FDR < 0.001 | 10.01 |
| Ccdc18 | 9.74E-11 | 0.88 | 1.76E-06 | FDR < 0.001 | 10.01 |
| Galm | 9.25E-11 | 1.348 | 1.67E-06 | FDR < 0.001 | 10.03 |
| Sfi1 | 5.44E-11 | 0.636 | 9.82E-07 | FDR < 0.001 | 10.26 |
| Klf15 | 5.40E-11 | 0.962 | 9.75E-07 | FDR < 0.001 | 10.27 |
| Ammecr1 | 4.69E-11 | 0.629 | 8.47E-07 | FDR < 0.001 | 10.33 |
| Slc25a48 | 3.59E-11 | 1.348 | 6.48E-07 | FDR < 0.001 | 10.45 |
| Hoxd9 | 3.29E-11 | 0.764 | 5.95E-07 | FDR < 0.001 | 10.48 |
| Sh3tc2 | 3.24E-11 | 0.934 | 5.85E-07 | FDR < 0.001 | 10.49 |
| Hopx | 2.83E-11 | 1.504 | 5.12E-07 | FDR < 0.001 | 10.55 |
| Acod1 | 2.30E-11 | 1.758 | 4.16E-07 | FDR < 0.001 | 10.64 |
| Pga5 | 2.25E-11 | 0.939 | 4.07E-07 | FDR < 0.001 | 10.65 |
| Ryr3 | 2.19E-11 | 1.212 | 3.95E-07 | FDR < 0.001 | 10.66 |
| Cacnb4 | 2.14E-11 | 0.915 | 3.86E-07 | FDR < 0.001 | 10.67 |
| Hsd11b2 | 2.09E-11 | 1.546 | 3.78E-07 | FDR < 0.001 | 10.68 |
| Pex11a | 2.08E-11 | 0.81 | 3.76E-07 | FDR < 0.001 | 10.68 |
| Slc5a11 | 1.92E-11 | 1.516 | 3.47E-07 | FDR < 0.001 | 10.72 |
| Fbln5 | 1.72E-11 | 0.809 | 3.10E-07 | FDR < 0.001 | 10.77 |
| Lims2 | 1.54E-11 | 0.99 | 2.79E-07 | FDR < 0.001 | 10.81 |
| Arl4d | 1.52E-11 | 1.08 | 2.75E-07 | FDR < 0.001 | 10.82 |
| Calca | 1.37E-11 | 1.447 | 2.47E-07 | FDR < 0.001 | 10.86 |
| Spon1 | 1.25E-11 | 1.029 | 2.25E-07 | FDR < 0.001 | 10.90 |
| Slain1 | 1.13E-11 | 0.989 | 2.04E-07 | FDR < 0.001 | 10.95 |
| Hp | 1.06E-11 | 1.792 | 1.92E-07 | FDR < 0.001 | 10.97 |
| Slc2a12 | 1.06E-11 | 1.374 | 1.91E-07 | FDR < 0.001 | 10.98 |
| Orai3 | 1.01E-11 | 0.534 | 1.82E-07 | FDR < 0.001 | 11.00 |
| Tnfrsf21 | 8.22E-12 | 1.052 | 1.48E-07 | FDR < 0.001 | 11.09 |
| Eng | 7.15E-12 | 0.776 | 1.29E-07 | FDR < 0.001 | 11.15 |
| Hdc | 6.84E-12 | 2.16 | 1.24E-07 | FDR < 0.001 | 11.16 |
| Gstm2 | 5.86E-12 | 1.635 | 1.06E-07 | FDR < 0.001 | 11.23 |
| Fam43a | 5.59E-12 | 1.169 | 1.01E-07 | FDR < 0.001 | 11.25 |
| 1700049E17Rik1 | 4.89E-12 | 0.86 | 8.84E-08 | FDR < 0.001 | 11.31 |
| Fhl1 | 4.64E-12 | 0.811 | 8.38E-08 | FDR < 0.001 | 11.33 |
| Stx18 | 3.85E-12 | 1.112 | 6.95E-08 | FDR < 0.001 | 11.41 |
| Vgll3 | 3.13E-12 | 0.915 | 5.65E-08 | FDR < 0.001 | 11.50 |
| Rims3 | 2.90E-12 | 1.43 | 5.24E-08 | FDR < 0.001 | 11.54 |
| Lsamp | 2.87E-12 | 0.775 | 5.19E-08 | FDR < 0.001 | 11.54 |
| Syn2 | 2.63E-12 | 1.495 | 4.75E-08 | FDR < 0.001 | 11.58 |
| Pcyt1b | 1.85E-12 | 0.984 | 3.34E-08 | FDR < 0.001 | 11.73 |
| Nexmif | 1.70E-12 | 0.766 | 3.07E-08 | FDR < 0.001 | 11.77 |
| Cd34 | 9.28E-13 | 1.226 | 1.68E-08 | FDR < 0.001 | 12.03 |
| Maob | 3.45E-13 | 1.42 | 6.24E-09 | FDR < 0.001 | 12.46 |
| Sult1a1 | 2.97E-13 | 0.996 | 5.37E-09 | FDR < 0.001 | 12.53 |
| Tgfbr3 | 2.95E-13 | 1.108 | 5.33E-09 | FDR < 0.001 | 12.53 |
| Ckb | 2.66E-13 | 1.665 | 4.81E-09 | FDR < 0.001 | 12.57 |
| Igf1 | 2.57E-13 | 1.702 | 4.65E-09 | FDR < 0.001 | 12.59 |
| Fads2 | 2.51E-13 | 1.765 | 4.53E-09 | FDR < 0.001 | 12.60 |
| 9130008F23Rik | 2.45E-13 | 0.625 | 4.42E-09 | FDR < 0.001 | 12.61 |
| St3gal4 | 2.42E-13 | 0.695 | 4.37E-09 | FDR < 0.001 | 12.62 |
| Sult1d1 | 2.15E-13 | 1.584 | 3.88E-09 | FDR < 0.001 | 12.67 |
| Ldhd | 1.40E-13 | 0.742 | 2.53E-09 | FDR < 0.001 | 12.85 |
| Cited4 | 1.11E-13 | 1.2 | 2.01E-09 | FDR < 0.001 | 12.95 |
| B3gnt5 | 5.06E-14 | 1.386 | 9.15E-10 | FDR < 0.001 | 13.30 |
| Hyal1 | 4.80E-14 | 1.401 | 8.68E-10 | FDR < 0.001 | 13.32 |
| S100g | 4.32E-14 | 1.937 | 7.81E-10 | FDR < 0.001 | 13.36 |
| Il17rb | 4.32E-14 | 1.965 | 7.80E-10 | FDR < 0.001 | 13.36 |
| Akap12 | 3.29E-14 | 0.858 | 5.94E-10 | FDR < 0.001 | 13.48 |
| Zmpste24 | 2.99E-14 | 1.396 | 5.41E-10 | FDR < 0.001 | 13.52 |
| Ano2 | 1.76E-14 | 1.746 | 3.17E-10 | FDR < 0.001 | 13.76 |
| Ctla2a | 9.31E-15 | 2.173 | 1.68E-10 | FDR < 0.001 | 14.03 |
| Tgfbrap1 | 8.36E-15 | 1.09 | 1.51E-10 | FDR < 0.001 | 14.08 |
| Scara5 | 2.30E-15 | 1.177 | 4.16E-11 | FDR < 0.001 | 14.64 |
| Atp1a2 | 1.53E-15 | 0.934 | 2.77E-11 | FDR < 0.001 | 14.81 |
| Cd248 | 1.32E-15 | 1.093 | 2.38E-11 | FDR < 0.001 | 14.88 |
| Klf9 | 6.80E-16 | 1.023 | 1.23E-11 | FDR < 0.001 | 15.17 |
| Ivns1abp | 3.17E-16 | 0.744 | 5.73E-12 | FDR < 0.001 | 15.50 |
| Zbtb16 | 9.26E-17 | 1.459 | 1.67E-12 | FDR < 0.001 | 16.03 |
| Npl | 2.67E-17 | 2.586 | 4.83E-13 | FDR < 0.001 | 16.57 |
| Abcb5 | 2.23E-17 | 1.849 | 4.03E-13 | FDR < 0.001 | 16.65 |
| Sgk1 | 4.81E-18 | 1.939 | 8.69E-14 | FDR < 0.001 | 17.32 |
| Fam189a2 | 1.50E-18 | 1.894 | 2.71E-14 | FDR < 0.001 | 17.82 |
| Glul | 5.15E-20 | 1.011 | 9.31E-16 | FDR < 0.001 | 19.29 |
| Plpp3 | 1.72E-20 | 1.32 | 3.11E-16 | FDR < 0.001 | 19.76 |
| Angptl7 | 4.92E-21 | 2.131 | 8.88E-17 | FDR < 0.001 | 20.31 |
| Jak2 | 4.59E-21 | 0.667 | 8.29E-17 | FDR < 0.001 | 20.34 |
| Spink1 | 8.90E-22 | 3.062 | 1.61E-17 | FDR < 0.001 | 21.05 |
| Col15a1 | 5.15E-22 | 1.562 | 9.29E-18 | FDR < 0.001 | 21.29 |
| Ovgp1 | 5.03E-22 | 1.952 | 9.08E-18 | FDR < 0.001 | 21.30 |
| Slc2a3 | 1.69E-27 | 3.008 | 3.06E-23 | FDR < 0.001 | 26.77 |
| Lrp2 | 5.86E-38 | 3.853 | 1.06E-33 | FDR < 0.001 | 37.23 |

**Supplementary Table 2 is listed separately due to its length**

**Supplementary Table 3. The sources of antibodies and RNAscope probes used in the present study**

| **REAGENT** | **SOURCE** | **IDENTIFIER** |
| --- | --- | --- |
| **Antibodies** | | |
| **Mouse anti β-actin** | **Santa Cruz** | **Cat# sc-47778** |
| **Rabbit anti KLF9 antibody** | **Abcam** | **Cat# ab227920** |
| **Rabbit anti KLF9 antibody** | **Abcam** | **Cat# ab26074** |
| **Rabbit anti COL6A5 antibody** | **Thermo Fisher** | **Cat# PA5-70781** |
| **Mouse anti COL7A1 antibody** | **Santa cruz** | **Cat# sc-53226** |
| **Rabbit anti COL17A1 antibody** | **Thermo Fisher** | **Cat# MA5-31984** |
| **Mouse anti IL-33 antibody** | **Biotechne** | **Cat# AF3626** |
| **Rabbit anti cytokeratin** | **DAKO** | **Cat# Z0622** |
| **Rabbit anti CD3 epsilon** | **Abcam** | **Cat# ab16669** |
| **Rabbit anti Cd8a** | **Invitrogen Life Sciences** | **Cat# PA5-114369** |
| **Rabbit anti Microglia (Iba1)** | **Biocare** | **Cat# CP 290** |
| **Rabbit anti CD11b** | **Novus Biologicals** | **Cat# NB110-89474** |
| **Rat anti CD45** | **BD Biosciences** | **Cat# 550539** |
| **RNAscope™ Probe- Mm-Il33** | **Advanced Cell Diagnostics** | **Cat# 400599** |
| **RNAscope™ Probe- Mm-Klf9** | **Advanced Cell Diagnostics** | **Cat# 488379** |
| **Rabbit anti Cleaved Caspase 3 (CC3)** | **Cell Signaling Technology** | **Cat# 9661** |
| **Anti-estrogen receptor a (rabbit polyclonal) antibodies** | **Santa Cruz** | **Cat# sc-542** |
| **Anti-progestrone receptor (rabbit polyclonal) antibodies** | **Dako** | **A0098** |
| **302** | **Made in Cheng’s lab** | **Monoclonal antibody recognizes TRα1PV mutant** |
| **C1** | **Made in Cheng’s lab** | **Monoclonal antibody recognizes wild-type TRα1 and TRβ1** |
| **C4** | **Made in Cheng’s lab** | **Monoclonal antibody recognizes wild-type TRα1 and TRβ1** |
| **T1** | **Made in Cheng’s lab** | **Rabbit polyclonal antibody recognizes TRα1PV mutant** |

**Supplementary Table 4. Primer sequences used in this study.**

| **Gene name** | **Forward sequence** | **Reverse sequence** |
| --- | --- | --- |
| ***Il33*** | TGGAGACTACAGCGCAAACT | TTATGGTGAGGCCAGAACGG |
| ***Il9r*** | GTGGACTCACAGTACCTGCC | AACGATACGGTCCTTGTGCC |
| ***Ccl3*** | AGGATACAAGCAGCAGCGAG | GAGCAAAGGCTGCTGGTTTC |
| ***Il23a*** | CAGCAGCTCTCTCGGAATCT | CTCCATGGGGCTATCAGGGA |
| ***Col6a5*** | GTGGATGAGTCAGTGGGGAC | ATTCAGACTGGTTGGCGCTT |
| ***Col7a1*** | ACTGTGGTTGCCCTCTATGC | TAGCACCTGGTACCCTCCTC |
| ***Col17a1*** | GAGATGGGTCCCTCCTGTCT | TAGTCAGACCCTCGGAGCAT |
| ***KLF9*** | TCTAGGGAAGGAAGACGCCA | GATGCCATTCACGTGCGTTT |
| ***Hr*** | CCCCTGTGAACGGCATTGT | CCCCTCCAAAAGGGAGCAG |
| ***Dio3*** | TGTACCTGACCACCGTTCA | GGTGCACCTTGTTGTAGTACTC |
| ***GAPDH*** | CATCACTGCCACCCAGAAGACTG | ATGCCAGTGAGCTTCCCGTTCAG |

**Supplementary Table 4. Primer sequences used in this study.**

| **Gene name** | **Forward sequence** | **Reverse sequence** |
| --- | --- | --- |
| ***Col6α5*** | GTGGATGAGTCAGTGGGGAC | ATTCAGACTGGTTGGCGCTT |
| ***Col7α1*** | ACTGTGGTTGCCCTCTATGC | TAGCACCTGGTACCCTCCTC |
| ***Col17α1*** | GAGATGGGTCCCTCCTGTCT | TAGTCAGACCCTCGGAGCAT |
| ***KLF9*** | TCTAGGGAAGGAAGACGCCA | GATGCCATTCACGTGCGTTT |
| ***Hr*** | CCCCTGTGAACGGCATTGT | CCCCTCCAAAAGGGAGCAG |
| ***Dio3*** | TGTACCTGACCACCGTTCA | GGTGCACCTTGTTGTAGTACTC |
| ***GAPDH*** | CATCACTGCCACCCAGAAGACTG | ATGCCAGTGAGCTTCCCGTTCAG |
